# Chemosensory and cardiometabolic improvements after a fasting-mimicking diet: A randomized cross-over clinical trial

**DOI:** 10.1016/j.xcrm.2025.101971

**Published:** 2025-02-18

**Authors:** Alessandro Micarelli, Simona Mrakic-Sposta, Alessandra Vezzoli, Sandro Malacrida, Sara Caputo, Beatrice Micarelli, Ilaria Misici, Valentina Carbini, Ilaria Iennaco, Ivan Granito, Valter D. Longo, Marco Alessandrini

**Affiliations:** 1 Unit of Neuroscience, Rehabilitation and Sensory Organs, UNITER ONLUS, Rome, Italy; 2 Institute of Clinical Physiology, National Research Council (CNR), Milan, Italy; 3 Institute of Mountain Emergency Medicine, Eurac Research, Bolzano, Italy; 4 European Longevity Institute, Milan, Italy; 5 Longevity Institute, Leonard Davis School of Gerontology, and Department of Biological Sciences, University of Southern California, Los Angeles, CA, USA; 6 University of Rome Tor Vergata – Department of Clinical Sciences and Translational Medicine – Ear-Nose-Throat Unit, Rome, Italy

**Keywords:** fasting-mimicking diet, smell, taste, obesity, leptin, ghrelin

## Abstract

Obesity is associated with a decrease in chemosensory perception acuity and increased disease risk, pointing to the need for feasible interventions that affect smell, taste, and cardiometabolic markers. Here, subjects with overweight/obesity are treated with six monthly cycles of a fasting-mimicking diet (FMD) lasting 5 days followed by a normal diet for the rest of the month to determine their effects on chemosensory function and cardiometabolic risk factors. Both arms of the 102 randomized cross-over trial participants indicate FMD-dependent improvements in a wide range of taste and smell chemosensory functions. The portion of hyposmic subjects is reduced from 38.1% at baseline to 6.4% at the end of 6 FMD cycles. FMD cycles also reduce cardiometabolic and inflammatory markers and drug use in diabetic patients. This trial provides evidence for the effect of periodic FMD cycles in improving chemosensory function while reducing cardiometabolic risk factors without requiring long-term lifestyle changes. The trial is registered at ClinicalTrials.gov (NCT04529161).

## Highlights

• Obesity is associated with a decrease in chemosensory perception acuity
• Obese participants followed six monthly cycles of a fasting-mimicking diet
• A fasting-mimicking diet reduces the number of hyposmic subjects from 38.1% to 6.4%
• A fasting-mimicking diet reduces cardiometabolic markers and diabetic drug use

## Graphical abstract

**Figure:**
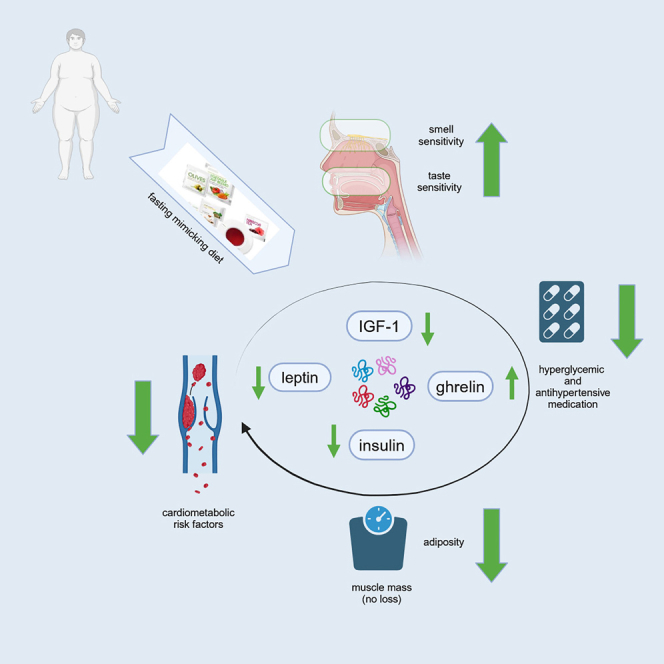

## Teaser

Micarelli et al. examine subjects with overweight/obesity treated with six monthly cycles of a fasting-mimicking diet (FMD) followed by a normal diet to determine their effects on chemosensory function and cardiometabolic risk factors. FMD improves a wide range of taste and smell functions and reduces cardiometabolic and inflammatory markers.

## Introduction

Obesity, one of the major risk factors for age-related diseases, has grown rapidly in the US, Europe, and many countries, yet efforts to reverse this epidemic have been largely unsuccessful, in part because long-term lifestyle changes are not sustainable for the majority of patients.^1^ Chemosensory perception—i.e., taste and smell—is a pivotal contributor of food palatability in humans that plays a significant role in food choice and energy consumption.^2^ In turn, nutrient intake or food habits may impact taste and smell sensitivity,^3^ since the tongue and olfactory bulb are obesity-associated organs, and their function is affected by the biochemical regulators promoting obesity.^4^ Because alterations in chemosensory perception can interfere with a healthy eating routine and lead to problems such as overconsumption of certain foods,^5^^,^^6^ a decrease in tastant perception has been associated with an increase in body mass index (BMI),^3^^,^^7^^,^^8^ and olfactory impairment is common in subjects with overweight (OW) and obesity.^2^^,^^9^^,^^10^ In addition, no study has evaluated the impact of dietary interventions that do not require daily lifestyle changes on olfactory performance, which is a concern considering that dietary non-compliance is a major limitation in managing obesity.^6^ Notably, some studies concluded that the reduced intake of sweet and energy-dense foods could lead to improvement in gustatory sensitivity.^3^ These aspects were partially corroborated by some short-term controlled interventional studies investigating taste perception after a period of a low-sugar diet.^11^^,^^12^^,^^13^

Periodic fasting-mimicking diets (FMDs) adopted for 5 days per month or less and providing 800–1,100 kcal per day have been tested for their potential role in reducing aging and disease risk factors.^14^ FMD cycles were shown to reduce glucose, insulin-like growth factor 1 (IGF-1), and insulin plasma levels, ameliorate lipid profile, decrease visceral fat, and modulate pro-inflammatory cytokines, particularly in subjects with high baseline levels of these markers.^15^^,^^16^^,^^17^^,^^18^ Notably, many of these factors, together with lower ghrelin and higher leptin serum levels, are collectively associated with a dampening effect on olfactory and gustatory performance in individuals with obesity.^4^^,^^7^^,^^9^^,^^10^^,^^19^

The aim of the present randomized, cross-over trial study was to test whether 6 monthly cycles of a 5-day periodic FMD, while allowing subjects to return to their normal diet for the rest of the month, may lead to improvements in olfactory and gustatory performance as well as in aging and disease risk factors/markers in individuals with OW and obesity.

## Results

**Figure 1:**
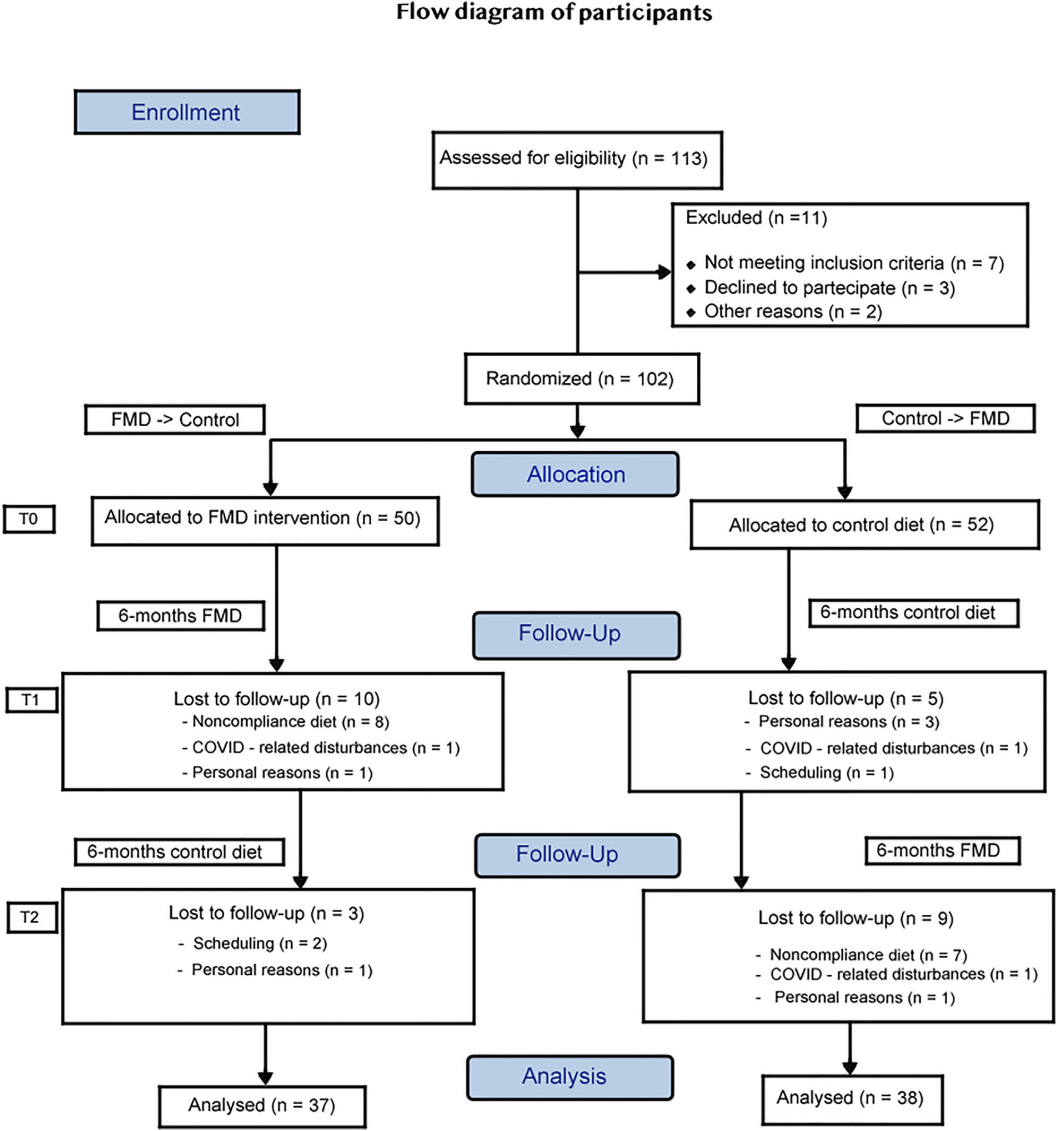
Consolidated Standards of Reporting Trials diagram of 113 subjects assessed for eligibility of which 102 were enrolled and randomized into the two arms of the study Participants in FMD->Control (*n* = 50) started the fasting-mimicking diet (FMD) after randomization. The FMD is provided for 5 days per month for six consecutive cycles. Control->FMD (*n* = 52) maintained their normal dietary habits for a 6-month monitoring period. After the first 6-month period, participants were crossed over to the FMD cycle regimen. Data were collected at enrollment (T0), after the first (T1) and second (T2) 6-month period. At the end of each FMD 6-month period, data were collected on average 5 days after subjects resumed their normal diet after the sixth FMD cycle.

**Figure 2:**
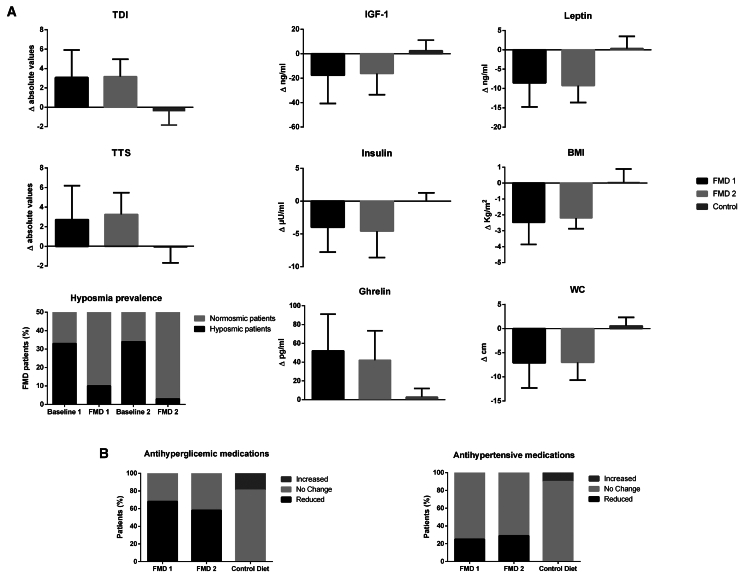
Impact of fasting-mimicking diet (A) Significant between-group (*p* value < 0.01) comparisons in chemosensory testing and biochemical assay differences (Δ) in all the participants before and after fasting-mimicking diet (FMD) intervention (*n* = 78, FMD 1 = 40 participants in FMD->Control and FMD 2 = 38 participants in Control->FMD) when compared to Control->FMD participants (*n* = 47) before and after the control 6-month period (Control). (B) Percentage of participants who respectively changed antidiabetic and antihypertensive medication administration after 6 monthly FMD cycles or control diet. All data are means ± SD. TDI, olfactory composite score; TTS, total taste score; ng, nanogram; mL, milliliter; %, percentage; FMD, fasting-mimicking diet; T0, baseline; T1, after 6 monthly FMD.

Eleven (51.6 ± 13.1 years; 5 females; BMI = 35.1 ± 3.8 kg/m^2^) out of 113 patients enrolled (53.1 ± 12.3 years; 58 females; BMI = 33.9 ± 4.1 kg/m^2^) between August 1, 2020, and March 31, 2022, were excluded; among 102 remaining participants, 50 and 52 participants were randomized to FMD->Control and Control->FMD group, respectively (Figure 1; Table 1). Non-adherence to all 6 cycles of the FMD intervention was reported in 16% (*n* = 8) and 14.8% (*n* = 7) of FMD->Control and Control->FMD arm participants, respectively (Figure 1). No differences were found in terms of baseline main parameters when comparing those participants who improved with those who worsened in terms of olfactory (composite olfactory score [TDI]) and gustatory (total taste score [TTS]) composite score after the FMD 6-month period and in TTS after the control period (Table 1). In contrast, participants who worsened in TDI after the control period were found to have significantly lower baseline BMI, weight, and waist circumference (WC) when compared to those who improved in TDI after the same period (see data in supplementary results—participants). According to previous studies reporting normative values for smell and taste dysfunction,^20^ none of the participants were found to be hypogeusic, whereas hyposmia was found at T0, T1, and T2 in 36% (18/50), 10% (4/40), and 13.5% (5/37), respectively, in the FMD->Control arm participants and in 32% (17/52), 40% (19/47), and 2.6% (1/38), respectively, in the Control->FMD participants. Thus, the total number of subjects with hyposmia immediately before beginning FMD cycles in arms 1 and 2 combined was reduced from 37 out of 97 or 38.1% to 5 out of 78 or 6.4% at the end of the 6 FMD cycles, a 5.9-fold decrease (Figure 2; Table 2). Notably, in the 6 months following the end of the FMD cycles in FMD->Control, only 1 patient worsened sufficiently to return to the hyposmia range.

**Table 1:** Socio-demographic aspects and characteristics of all participants at baseline

	FMD->Control baseline (*n =* 50)	Control->FMD baseline (*n =* 52)	*p* value
Age (years)	54.4 ± 12.3	52.3 ± 12.1	*p* = 0.39
Gender	24 females, 26 males	29 females, 23 males	*p* = 0.43
Education (years)	14.2 ± 4.2	13.6 ± 3.7	*p* = 0.45

**Obesity stages**			

Overweight (25 ≤ BMI ≤ 29.9)	8 (16)	9 (17.3)	*p* = 0.75
Stage I obesity (30 ≤ BMI ≤ 34.9)	25 (50)	22 (42.3)	
Stage II obesity (35 ≤ BMI ≤ 39.9)	12 (24)	17 (32.6)	
Stage III obesity (BMI ≥ 40)	5 (10)	4 (7.6)	

**History of**			

Hypertension	29 (58)	32 (61.5)	*p* = 0.85
Arthrosis	23 (46)	19 (36.5)	*p* = 0.53
Thyroid nodules	9 (18)	6 (11.5)	*p* = 0.42
Coronary heart disease	7 (14)	6 (11.5)	*p* = 0.74
Type 2 diabetes	21 (42)	22 (42.3)	*p* = 0.98
Psoriasis	1 (2)	0 (0)	–
OSAS	13 (26)	15 (28.8)	*p* = 0.8

**Medication**			

Metformin	21 (42)	22 (42.3)	*p* = 0.98
ACEIs/ARBs	20 (40)	24 (46.1)	*p* = 0.69
Loop diuretics	6 (12)	5 (9.6)	*p* = 0.72
Calcium antagonists	13 (26)	14 (26.9)	*p* = 0.93
Thiazide diuretics	7 (14)	9 (17.3)	*p* = 0.69
NSAIDs	23 (46)	25 (48)	*p* = 0.89
PPIs	26 (52)	24 (46.1)	*p* = 0.72
Statin	18 (36)	16 (30.7)	*p* = 0.69

**Chemosensory testing**			

OT	7.49 ± 2.74	7.25 ± 1.69	*p* = 0.6
OD	11.28 ± 1.73	11.71 ± 2.12	*p* = 0.26
OI	12.58 ± 1.59	12.78 ± 1.14	*p* = 0.44
TDI	31.25 ± 3.74	31.75 ± 3.42	*p* = 0.47
Sweet	6.64 ± 1.28	6.71 ± 1.31	*p* = 0.7
Sour	4.9 ± 1.65	4.88 ± 1.26	*p* = 0.95
Salty	5.78 ± 1.56	5.69 ± 1.65	*p* = 0.78
Bitter	5.62 ± 1.57	5.73 ± 1.72	*p* = 0.73
TTS	22.94 ± 4.1	23.01 ± 3.97	*p* = 0.92

**Biochemical assays**			

Leptin (ng/mL)	26.89 (21.8–29.67)	26.31 (20.13–34.87)	*p* = 0.28
Ghrelin (pg/mL)	197.13 ± 42.13	203.86 ± 37.74	*p* = 0.39
IGF-1 (ng/mL)	159.51 ± 23.75	157.06 ± 26.38	*p* = 0.62
Serum glucose (mg/dL)	99 (92–105.75)	99 (88.75–111)	*p* = 0.59
Insulin (μU/mL)	11.49 (4.96–15.06)	10.61 (8.6–15.7)	*p* = 0.25
Total cholesterol (mg/dL)	223.28 ± 38.36	219.59 ± 36.3	*p* = 0.61
LDL (mg/dL)	142.32 ± 37.93	140.78 ± 35.27	*p* = 0.83
HDL (mg/dL)	55 (43.75–66.5)	52 (46–65.25)	*p* = 0.4
TGs (mg/dL)	111 (82.25–158.5)	115 (95.75–137)	*p* = 0.69
Conjugated bilirubin (mg/dL)	0.16 ± 0.06	0.18 ± 0.11	*p* = 0.22
Unconjugated bilirubin (mg/dL)	0.42 ± 0.19	0.42 ± 0.22	*p* = 0.97
ESR (mm/h)	12 (5–16.75)	11 (6–16.25)	*p* = 0.73
CRP (mg/L)	0.9 (0.3–2.99)	1.76 (0.38–3)	*p* = 0.79
AST (U/L)	23.32 ± 8.42	23.25 ± 7.18	*p* = 0.96
ALT (U/L)	25.72 ± 11.85	28.32 ± 13.56	*p* = 0.83
Uraemia (mg/dL)	35.36 ± 12.78	35.17 ± 9.29	*p* = 0.93
Serum creatinine (mg/dL)	0.84 ± 0.15	0.84 ± 0.15	*p* = 0.95
HOMA %B	102.97 ± 34.11	110.95 ± 45.34	*p* = 0.31
HOMA %S	76.68 ± 34.65	76.15 ± 40.96	*p* = 0.94
HOMA-IR	1.56 ± 0.65	1.71 ± 0.96	*p* = 0.36

**Anthropometric variables**			

WC (cm)	111.68 ± 10.38	112.18 ± 9.6	*p* = 0.8
Weight (kg)	94.57 ± 14.5	97.07 ± 14.44	*p* = 0.38
BMI (kg/m^2^)	33.7 ± 4.38	33.87 ± 3.91	*p* = 0.83
FM%∗	39.72 ± 8.25	40.95 ± 7.32	*p* = 0.42
FM (kg)∗	37.41 ± 9.47	39.7 ± 9.11	*p* = 0.21
MM%∗	26.51 ± 4.33	25.9 ± 4.24	*p* = 0.47
MM (kg)∗	25.16 ± 5.9	25.22 ± 5.86	*p* = 0.96
VF level∗	15.38 ± 4.93	14.4 ± 4.68	*p* = 0.3

Values are given in mean ± standard deviation (SD) and ±95% confidence interval (CI) for normally distributed variables or median (Q1–Q3) for log-normally distributed variables and frequencies *n* (%) for categorical variables. BMI, body mass index; OSAS, obstructive sleep apnea syndrome; ACEIs, angiotensin-converting enzyme inhibitors; ARBs, angiotensin II receptor blockers; NSAIDs, non-steroidal anti-inflammatory drugs; PPIs, proton-pump inhibitors. OT, odor threshold; OD, odor discrimination; OI, odor identification; and their sum (TDI); TTS, total taste score; ALT, alanine aminotransferase; AST, aspartate aminotransferase; TGs, triglycerides; HDL, high-density lipoprotein cholesterol; LDL, low-density lipoprotein cholesterol; ESR, erythrocyte sedimentation rate; CRP, C-reactive protein; HOMA %B, steady-state beta cell function; HOMA %S, insulin sensitivity; HOMA-IR, homeostasis model assessment of insulin resistance; WC, waist circumference; BMI, body mass index; ng, nanogram; pg, picogram; mL, milliliter; mg, milligram; dL, deciliter; U, international unit; μU, micro international unit; mm, millimeter; L, liter; h, hour; cm, centimeter; m, meter; kg, kilogram; %, percentage; FM, fat mass; MM, muscle mass; X^2^, chi-square. *p* values < 0.01 were considered significant. ∗, estimated by means of bioelectrical impedance analysis.

**Table 2:** Changes from baseline in chemosensory testing by means of per-protocol and intention-to-treat analysis

		Baseline	CTRL: 6 months after baseline/FMD: 5 days after sixth FMD cycle			
		Mean ± SD (CI)	Mean ± SD (CI)	*p* ƚ	Difference: Δ Mean ± SD	Efficacy (comparing Δ)§*p*
**Per-protocol analysis**						

**Hyposmic patients**	FMD 1	18/50 (36%)	4/40 (10/%)	–		
	FMD 2	19/47 (40%)	1/38 (2.6%)			
	control diet	17/52 (32%)	19/47 (40%)			
**TDI**	FMD 1 (*n* = 40)	31.18 ± 3.6 (30.06–32.29)	34.25 ± 3.46 (33.17–35.32)	<0.001	3.11 ± 2.37	<0.001
	FMD 2 (*n* = 38)	31.51 ± 2.86 (30.6–32.42)	34.68 ± 2.86 (33.77–35.59)	<0.001		
	control diet (*n* = 47)	31.54 ± 3.45 (30.55–32.52)	31.12 ± 2.98 (30.27–31.98)	0.53	−0.41 ± 1.42	
**TTS**	FMD 1 (*n* = 40)	22.55 ± 4.08 (21.28–23.81)	25.27 ± 4.06 (24.01–26.53)	0.003	2.98 ± 2.92	<0.001
	FMD 2 (*n* = 38)	22.68 ± 3.63 (21.52–23.84	25.94 ± 3.52 (24.82–27.06)	<0.001		
	control diet (*n* = 47)	23.02 ± 4.14 (21.83–24.2)	22.95 ± 3.75 (21.88–24.03)	0.93	−0.06 ± 1.6	
**OT**	FMD 1 (*n* = 40)	7.58 ± 2.42 (6.83–8.33)	8.98 ± 2.03 (8.35–9.61)	0.006	1.45 ± 1.08	<0.001
	FMD 2 (*n* = 38)	7.11 ± 1.48 (6.64–7.58)	8.63 ± 1.71 (8.08–9.17)	<0.001		
	control diet (*n* = 47)	7.15 ± 1.65 (6.68–7.63)	7.06 ± 1.49 (6.63–7.49)	0.76	−0.09 ± 0.56	
**OD**	FMD 1 (*n* = 40)	10.97 ± 1.6 (10.47–11.47)	11.97 ± 1.64 (11.46–12.48)	0.007	1.06 ± 1.2	<0.001
	FMD 2 (*n* = 38)	11.73 ± 1.81 (11.16–12.31)	12.86 ± 1.39 (12.42–13.31)	0.003		
	control diet (*n* = 47)	11.55 ± 2.16 (10.93–12.17)	11.57 ± 1.82 (11.05–12.09)	0.95	0.02 ± 0.92	
**OI**	FMD 1 (*n* = 40)	12.75 ± 1.62 (12.24–13.25)	13.25 ± 1.62 (12.74–13.75)	0.17	0.51 ± 0.92	<0.001
	FMD 2 (*n* = 38)	12.65 ± 1.25 (12.25–13.05)	13.18 ± 1.22 (12.79–13.57)	0.06		
	control diet (*n* = 47)	12.82 ± 1.1 (12.51–13.14)	12.48 ± 1.23 (12.13–12.84)	0.16	−0.34 ± 0.93	
**Sweet**	FMD 1 (*n* = 40)	6.52 ± 1.32 (6.11–6.93)	7.37 ± 0.89 (7.09–7.65)	0.001	0.91 ± 0.98	<0.001
	FMD 2 (*n* = 38)	6.63 ± 0.94 (6.33–6.93)	7.6 ± 0.78 (7.35–7.85)	<0.001		
	control diet (*n* = 47)	6.8 ± 1.32 (6.42–7.18)	6.63 ± 1 (6.34–6.92)	0.48	−0.17 ± 0.81	
**Sour**	FMD 1 (*n* = 40)	4.85 ± 1.59 (4.35–5.34)	5.67 ± 1.28 (5.27–6.07)	0.012	0.88 ± 0.95	<0.001
	FMD 2 (*n* = 38)	5.02 ± 1.02 (4.69–5.35)	5.97 ± 1.05 (5.63–6.3)	<0.001		
	control diet(*n* = 47)	4.8 ± 1.26 (4.44–5.16)	5.06 ± 1.09 (4.75–5.37)	0.29	0.25 ± 0.67	
**Salty**	FMD 1 (*n* = 40)	5.65 ± 1.4 (5.21–6.08)	6.17 ± 1.37 (5.74–6.6)	0.09	0.52 ± 1.07	0.004
	FMD 2 (*n* = 38)	5.52 ± 1.53 (5.03–6.01)	6.05 ± 1.33 (5.62–6.47)	0.11		
	control diet (*n* = 47)	5.68 ± 1.6 (5.22–6.13)	5.65 ± 1.56 (5.21–6.1)	0.94	−0.02 ± 0.89	
**Bitter**	FMD 1 (*n* = 40)	5.52 ± 1.53 (5.04–6)	6.32 ± 1.28 (5.92–6.72)	0.013	0.8 ± 1.05	<0.001
	FMD 2 (*n* = 38)	5.5 ± 1.57 (5–5.99)	6.31 ± 1.33 (5.89–6.74)	0.017		
	control diet (*n* = 47)	5.72 ± 1.72 (5.22–6.21)	5.59 ± 1.59 (5.13–6.05)	0.71	−0.12 ± 0.84	

Intention-to-treat analysis						
		Baseline (FMD 1 = 50; FMD 2 = 47; control diet = 52)	CTRL: 6 months after baseline/FMD: 5 days after sixth FMD cycle (FMD 1 = 40; FMD 2 = 38; control diet = 47)	–	–	–
		Mean ± SD (CI)	Mean ± SD (CI)	*p* ƚ	Difference: Δ Mean ± SD	Efficacy (comparing Δ)§*p*
**TDI**	FMD 1	31.25 ± 3.74 (30.21–32.29)	34.25 ± 3.46 (33.17–35.32)	<0.001	2.38 ± 2.46	<0.001
	FMD 2	31.12 ± 2.98 (30.27–31.98)	34.68 ± 2.86 (33.77–35.59)	<0.001		
	control diet, arm 2	31.75 ± 3.42 (30.82–32.69)	31.12 ± 2.98 (30.27–31.98)	0.33	−0.37 ± 1.35	
**TTS**	FMD 1	22.94 ± 4.1 (21.8–24.07)	25.27 ± 4.06 (24.01–26.53)	0.008	2.28 ± 2.85	<0.001
	FMD 2	22.95 ± 3.75 (21.88–24.03)	25.94 ± 3.52 (24.82–27.06)	<0.001		
	control diet, arm 2	23.01 ± 3.97 (21.93–24.1)	22.95 ± 3.75 (21.88–24.03)	0.93	−0.05 ± 1.52	
**OT**	FMD 1	7.49 ± 2.74 (6.73–8.25)	8.98 ± 2.03 (8.35–9.61)	0.005	1.11 ± 1.12	<0.001
	FMD 2	7.06 ± 1.49 (6.63–7.49)	8.63 ± 1.71 (8.08–9.17)	<0.001		
	control diet, arm 2	7.25 ± 1.69 (6.8–7.71)	7.06 ± 1.49 (6.63–7.49)	0.54	−0.08 ± 0.53	
**OD**	FMD 1	11.28 ± 1.73 (10.79–11.76)	11.97 ± 1.64 (11.46–12.48)	0.056	0.81 ± 1.14	<0.001
	FMD 2	11.57 ± 1.82 (11.05–12.09 =	12.86 ± 1.39 (12.42–13.31)	<0.001		
	control diet, arm 2	11.71 ± 2.12 (11.13–12.28)	11.57 ± 1.82 (11.05–12.09)	0.73	0.01 ± 0.87	
**OI**	FMD 1	12.58 ± 1.59 (12.13–13.02)	13.25 ± 1.62 (12.74–13.75)	0.052	0.39 ± 0.83	<0.001
	FMD 2	12.48 ± 1.23 (12.13–12.84)	13.18 ± 1.22 (12.79–13.57)	0.011		
	control diet, arm 2	12.78 ± 1.14 (12.47–13.09)	12.48 ± 1.23 (12.13–12.84)	0.21	−0.3 ± 0.89	
**Sweet**	FMD 1	6.64 ± 1.28 (6.28–6.99)	7.37 ± 0.89 (7.09–7.65)	0.002	0.69 ± 0.94	<0.001
	FMD 2	6.63 ± 1 (6.34–6.92)	7.6 ± 0.78 (7.35–7.85)	<0.001		
	control diet, arm 2	6.71 ± 1.31 (6.35–7.06)	6.63 ± 1 (6.34–6.92)	0.75	−0.15 ± 0.77	
**Sour**	FMD 1	4.9 ± 1.65 (4.44–5.35)	5.67 ± 1.28 (5.27–6.07)	0.017	0.67 ± 0.91	0.002
	FMD 2	5.06 ± 1.09 (4.75–5.37)	5.97 ± 1.05 (5.63–6.3)	<0.001		
	control diet, arm 2	4.88 ± 1.26 (4.54–5.22)	5.06 ± 1.09 (4.75–5.37)	0.45	0.23 ± 0.64	
**Salty**	FMD 1	5.78 ± 1.56 (5.34–6.21)	6.17 ± 1.37 (5.74–6.6)	0.21	0.4 ± 0.96	0.008
	FMD 2	5.65 ± 1.56 (5.21–6.01)	6.05 ± 1.33 (5.62–6.47)	0.22		
	control diet, arm 2	5.69 ± 1.65 (5.24–6.14)	5.65 ± 1.56 (5.21–6.1)	0.91	−0.01 ± 0.85	
**Bitter**	FMD 1	5.62 ± 1.57 (5.18–6.05)	6.32 ± 1.28 (5.92–6.72)	0.024	0.61 ± 0.98	<0.001
	FMD 2	5.59 ± 1.59 (5.13–6.05)	6.31 ± 1.33 (5.89–6.74)	0.029		
	control diet, arm 2	5.73 ± 1.72 (5.26–6.2)	5.59 ± 1.59 (5.13–6.05)	0.68	−0.11 ± 0.8	

Changes in chemosensory testing by means of per-protocol and intention-to-treat analysis. FMD 1, FMD->Control; FMD 2, Control->FMD. OT, odor threshold; OD, odor discrimination; OI, odor identification; and their sum (TDI); TTS, total taste score. Values are given in mean ± standard deviation (SD) and ±95% confidence interval (CI) for normally distributed variables. ƚ *p* values comparing within-group changes were calculated using paired two-tailed Student’s t test (*p* values < 0.01 were considered significant). **§** comparisons between the combination of pre-post FMD differences (Δ) in FMD 1 and FMD 2 (*n* = 78) participants and Control->FMD T0-T1 (Control diet) Δ values (*n* = 47) participants performed using two-tailed two-sample t tests (*p* values < 0.01 were considered significant).

Socio-demographic aspects and between-group comparison at baseline—highlighting no significant differences between arms—are depicted in Table 1.

### Changes in chemosensory testing

#### Changes from baseline

**Figure 3:**
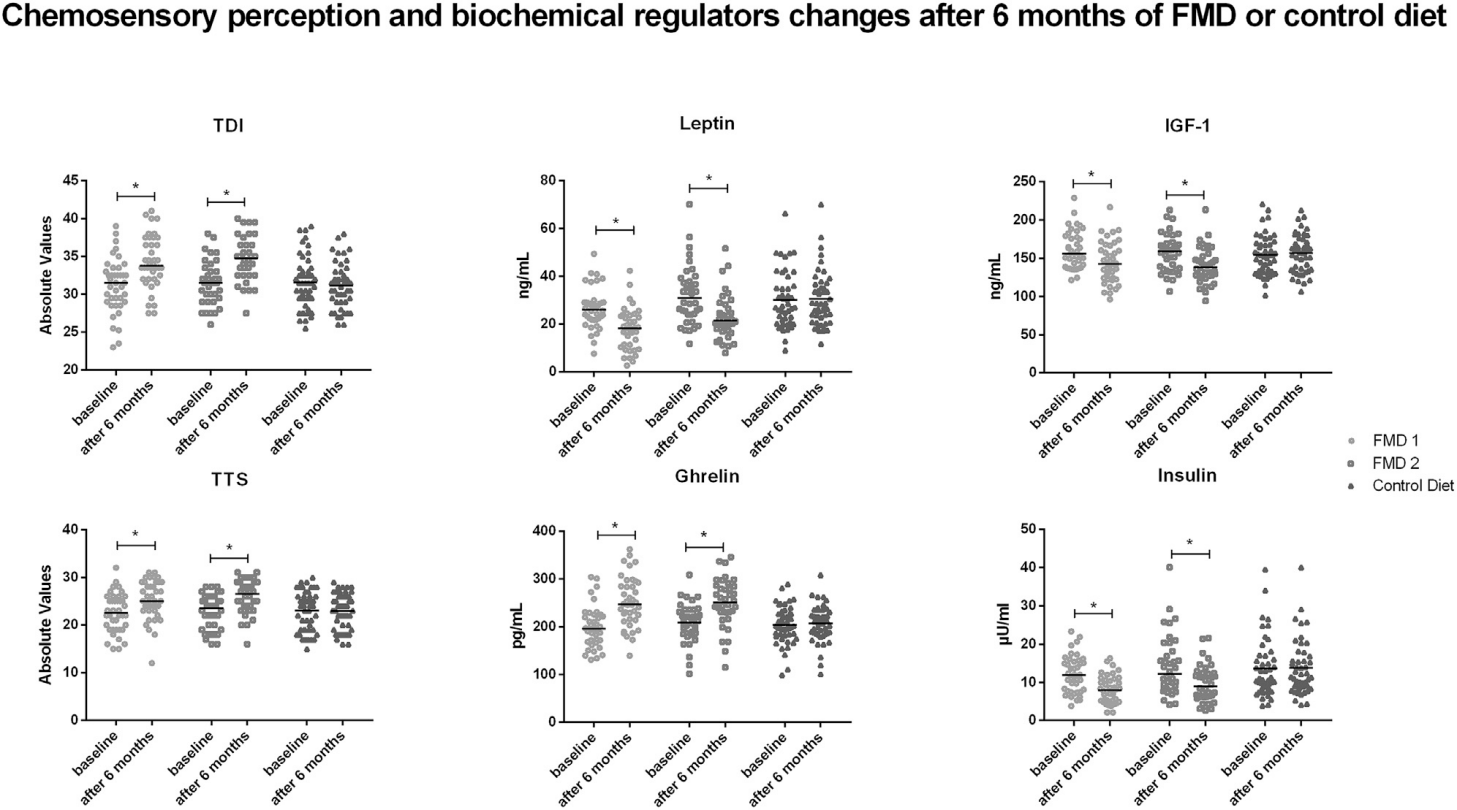
Scatter dot plot (line at median) depicting changes from baseline in main chemosensory perception testing and biochemical regulators at baseline and after 6 months of control diet (*n* = 47) or fasting-mimicking diet in FMD->Control (FMD 1, *n* = 40) and Control->FMD (FMD 2, *n* = 38) participants Asterisks indicate significant within-subject differences (exact *p* values are given in the text). ng, nanogram; pg, picogram; mL, milliliter; μU, micro international unit; TTS, total taste score; TDI, composite olfactory score. Exact *p* values are given in the text.

After the 6 FMD cycles, the within-group analysis in the FMD->Control arm (*n* = 40) showed a significant (*p* < 0.01) increase in odor threshold (OT), odor discrimination (OD), TDI, TTS, and sweet (Table 2; Figure 3), whereas no significant differences were found in the Control->FMD arm (*n* = 47) when comparing T0 with T1 (control diet period). In contrast, at T2 compared to T1 (*n* = 38), the impact of FMD cycles in the Control->FMD arm was similar to that found in the FMD->Control arm with a significant (*p* < 0.01) increase in OT, OD, TDI, TTS, sweet, and sour (Table 2; Figure 3). A significant (*p* < 0.01) increase between groups ΔOT, ΔOD, Δodor identification (OI), ΔTDI, Δsweet, Δsour, Δbitter, Δsalty, and ΔTTS was found in the combined FMD groups after 6 FMD cycles (FMD->Control + Control->FMD) compared to the control group after the first 6-month period (control diet) (Table 2; Figure 2).

In agreement with the lack of differences between arms for many markers at T2, some of chemosensory testing variables were found to be significantly (*p* < 0.01) different when also comparing FMD->Control T2 with FMD->Control T0 (*n* = 37) values: TDI, TTS, and sweet, whereas no significant (*p* > 0.01) differences were found in OT, OD, OI, sour, salty, and bitter. At T2, after FMD->Control participants returned to their dietary habits for 6 months, no significant (*p* > 0.01) differences were found in OT, OD, OI, TDI, sweet, sour, bitter, salty, and TTS when compared to T1. These results indicate a partial washout effect of switching from the FMD to a control diet for 6 months.

When comparing Control->FMD T2 with Control->FMD T0 (baseline), similar results were found confirming a significant (*p* < 0.01) increase in OT, OD, TDI, TTS, sweet, and sour, but not (*p* > 0.01) in OI, salty, and bitter, again in agreement with a partial washout effect after the return to a 6-month control diet.

#### Between-group analysis

**Figure 4:**
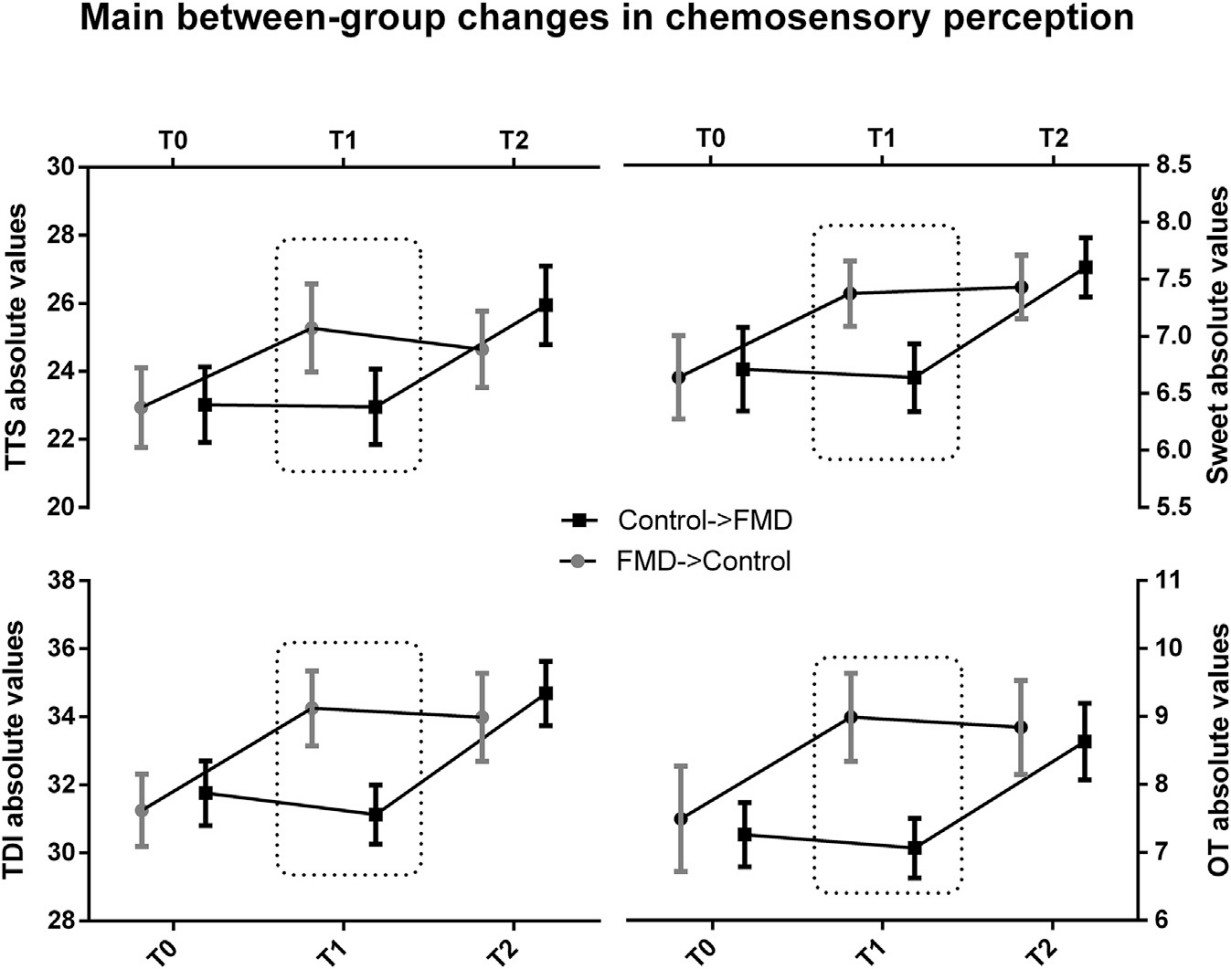
Main between-group changes in chemosensory perception testing at baseline (T0), after the first semester (T1) in which FMD->Control participants followed the fasting-mimicking diet intervention and Control->FMD participants maintained their regular eating habits and after the second semester (T2) in which FMD->Control and 2 were crossed over Dotted boxes indicate significant between-group differences (exact *p* values are given in the text). Values are given in mean with 95% CI. OT, odor threshold; TTS, total taste score; TDI, composite olfactory score.

The between-group analysis found that—after 6 monthly FMD cycles—FMD->Control participants demonstrated a significant (*p* < 0.016) increase in OT, OI, and TDI as well as in sweet and TTS when compared with the Control->FMD group at the end of the control diet period. No significant (*p* > 0.016) between-group changes were found in OD and salty, although a trend for improvements was observed for sour (*p* = 0.018) and bitter (*p* = 0.02) (Table 3; Figure 4). When comparing the Control->FMD arm at the end of 6 FMD cycles after the cross-over (T2) with the FMD->control arm at the end of the 6-month washout period (T2), only OD was found to be significantly higher in Control->FMD (12.86 ± 1.39) with respect to FMD->Control (11.87 ± 1.83) participants (*p* = 0.01). At the same T2 time point, no significant (*p* > 0.016) differences were found in OT, OI, TDI, sweet, sour, bitter, salty, and TTS (Table 3; Figure 4). These results again support the long-lasting effects of many but not all of the improvements caused by FMD cycles. The interactions for TDI and TTS with time points and arms were, respectively, F(2, 140) = 19.979, *p* < 0.001 and F(2, 140) = 16.534, *p* < 0.001. However, Bonferroni correction confirmed as significant (*p* < 0.016) only the comparisons between T0 and T1 and T0 and T2 in FMD->Control and between T0 and T2 and T1 and T2 in Control->FMD participants. No significant effect (*p* > 0.016) related to the arms was found, thus highlighting the main effect of interaction as explained by the significant differences across time points and not by the allocation arms and minimizing the likelihood of a carryover effect on main outcome changes. Weak negative and positive correlations were respectively found between weight loss and Δsweet (R = −0.23, *p* = 0.03) and Δsour (R = 0.22, *p* = 0.04) (Table S1).

**Table 3:** Between-group comparisons in chemosensory testing in all participants at T0, T1 and T2

	T0		p	T1		p	T2		*p*
	FMD->Control (*n* = 50)	Control->FMD (*n =* 52)		FMD->Control (*n* = 40)	Control->FMD (*n* = 47)		FMD->Control (*n* = 37)	Control->FMD (*n* = 38)	
	Mean ± SD (CI)	Mean ± SD (CI)		Mean ± SD (CI)	Mean ± SD (CI)		Mean ± SD (CI)	Mean ± SD (CI)	
OT	7.49 ± 2.74 (6.73–8.25)	7.25 ± 1.69 (6.8–7.71)	0.6	8.98 ± 2.03 (8.35–9.61)	7.06 ± 1.49 (6.63–7.49)	<0.001	8.83 ± 2.06 (8.17–9.5)	8.63 ± 1.71 (8.08–9.17)	0.63
OD	11.28 ± 1.73 (10.79–11.76)	11.71 ± 2.12 (11.13–12.28)	0.26	11.97 ± 1.64 (11.46–12.48)	11.57 ± 1.82 (11.05–12.09)	0.28	11.87 ± 1.83 (11.28–12.46)	12.86 ± 1.39 (12.42–13.31)	0.01
OI	12.58 ± 1.59 (12.13–13.02)	12.78 ± 1.14 (12.47–13.09)	0.44	13.25 ± 1.62 (12.74–13.75)	12.48 ± 1.23 (12.13–12.84)	0.015	13.27 ± 1.5 (12.78–13.75)	13.18 ± 1.22 (12.79–13.57)	0.78
TDI	31.25 ± 3.74 (30.21–32.29)	31.75 ± 3.42 (30.82–32.69)	0.47	34.25 ± 3.46 (33.17–35.32)	31.12 ± 2.98 (30.27–31.98)	<0.001	33.98 ± 3.86 (32.74–35.23)	34.68 ± 2.86 (33.77–35.59)	0.37
Sweet	6.64 ± 1.28 (6.28–6.99)	6.71 ± 1.31 (6.35–7.06)	0.7	7.37 ± 0.89 (7.09–7.65)	6.63 ± 1 (6.34–6.92)	<0.001	7.43 ± 0.83 (7.16–7.7)	7.6 ± 0.78 (7.35–7.85)	0.35
Sour	4.9 ± 1.65 (4.44–5.35)	4.88 ± 1.26 (4.54–5.22)	0.95	5.67 ± 1.28 (5.27–6.07)	5.06 ± 1.09 (4.75–5.37)	0.018	5.56 ± 1.28 (5.15–5.98)	5.97 ± 1.05 (5.63–6.3)	0.13
Salty	5.78 ± 1.56 (5.34–6.21)	5.69 ± 1.65 (5.24–6.14)	0.78	6.17 ± 1.37 (5.74–6.6)	5.65 ± 1.56 (5.21–6.1)	0.1	5.89 ± 1.3 (5.47–6.31)	6.05 ± 1.33 (5.62–6.47)	0.6
Bitter	5.62 ± 1.57 (5.18–6.05)	5.73 ± 1.72 (5.26–6.2)	0.73	6.22 ± 1.59 (5.92–6.72)	5.59 ± 1.59 (5.13–6.05)	0.02	5.83 ± 1.28 (5.42–6.25)	6.31 ± 1.33 (5.89–6.74)	0.11
TTS	22.94 ± 4.1 (21.8–24.07)	23.01 ± 3.97 (21.93–24.1)	0.92	25.27 ± 4.06 (24.01–26.53)	22.95 ± 3.75 (21.88–24.03)	0.007	24.64 ± 3.36 (23.56–25.73)	25.94 ± 3.52 (24.82–27.06)	0.1

Between-group comparisons in chemosensory testing in all participants at T0, T1, and T2. OT, odor threshold; OD, odor discrimination; OI, odor identification; and their sum (TDI); TTS, total taste score. Values are given in mean ± standard deviation (SD) and ±95% confidence interval (CI).

### Changes in cardiometabolic and anthropometric variables

#### Changes from baseline

After 6 FMD cycles, in the within-group analysis, significant (*p* < 0.01) decreases were found in the FMD->Control arm (*n* = 40) in leptin, IGF-1, total cholesterol, low-density lipoprotein (LDL), serum glucose, insulin, insulin sensitivity (homeostasis model assessment [HOMA] %S), HOMA of insulin resistance (HOMA-IR), aspartate aminotransferase (AST), alanine aminotransferase (ALT), WC, estimated fat mass (FM), and visceral fat (VF) level with a significant increase in ghrelin and estimated muscle mass (MM) (Omron HBF-500 BIA, Omron Medizintechnik, Mannheim, Germany) compared to T0 baseline (Table S2; Figure 3). Similar changes were also found when comparing Control->FMD participants (*n* = 38) T2 with T1, whereas no significant (*p* > 0.01) differences were found in the control diet period in the Control->FMD arm (*n* = 47) when comparing T1 and T0 (Table S2; Figure 3).

The comparison between changes in all the participants after FMD cycles combined (FMD->Control + Control->FMD) and Control->FMD participants at the end of the control diet (control diet T1) highlighted a significant (*p* < 0.01) decrease in leptin, IGF-1, total cholesterol, LDL, triglycerides (TGs), erythrocyte sedimentation rate (ESR), C-reactive protein (CRP), serum glucose, insulin, HOMA-IR, AST, ALT, uremia, and serum creatinine, whereas a significant (*p* < 0.01) increase was found in ghrelin and HOMA %S (Table S2; Figure 2). Finally, a significant (*p* < 0.01) decrease in WC, weight, BMI, FM (% and kg), and VF level and a significant (*p* < 0.01) increase in MM were found in the same comparison (Table S2).

The following variables were found to be significantly (*p* < 0.01) different when also comparing T2 with T0 values for FMD->Control participants (*n* = 37): leptin, ghrelin, total cholesterol, insulin, ALT, HOMA %S, HOMA-IR, estimated FM (in both % and kg), and estimated MM%. No significant (*p* > 0.01) differences were found in IGF-1, high-density lipoprotein (HDL), LDL, TGs, AST, ESR, CRP, serum glucose, conjugated and unconjugated bilirubin, uremia, serum creatinine, steady-state beta cell function (HOMA %B), weight, WC, BMI, estimated MM kg, and VF level in the same comparison. After the second 6-month period in which FMD->Control participants returned to their dietary habits (T2), compared to T1 values, no significant (*p* > 0.01) differences were found in leptin, IGF-1, ghrelin, serum glucose, insulin, total cholesterol, LDL, HDL, TGs, ESR, CRP, conjugated and unconjugated bilirubin, AST, ALT, uremia and serum creatinine, HOMA %B, HOMA %S, HOMA-IR, WC, weight, BMI, and VF level. A significant (*p* < 0.01) increase and decrease, respectively, in estimated FM (in both % and kg) and MM (in both % and kg, respectively) were found. Again, these results indicate that many of the effects of FMD cycles are lasting at least 6 months but many do not.

When comparing Control->FMD (*n* = 38) after 6 FMD cycles following the cross-over (T2 vs. T1), we observed a significant (*p* < 0.01) decrease in leptin, IGF-1, serum glucose, insulin, total cholesterol, LDL, AST, ALT and uremia, HOMA-IR, as well as in WC, BMI, estimated FM (in both % and kg), and VF levels, whereas a significant (*p* < 0.01) increase was found in ghrelin, HDL, HOMA %S, and estimated MM (in both % and kg, respectively). No significant (*p* > 0.01) differences were found in TGs, ESR, CRP, conjugated and unconjugated bilirubin, serum creatinine, HOMA %B, and weight (Table S2; Figure 3). Many of these parameters were found to be significantly (*p* < 0.01) decreased when also comparing T2 to T0 in this Control->FMD: leptin, serum glucose, insulin, total cholesterol, LDL, AST, ALT and uremia, HOMA-IR, as well as WC, BMI, estimated FM (in both % and kg), and VF levels, whereas a significant (*p* < 0.01) increase in ghrelin, HOMA %S, and estimated MM (in both % and kg) was found. No significant (*p* > 0.01) differences were found in TGs, ESR, CRP, conjugated and unconjugated bilirubin, serum creatinine, HOMA %B, weight, IGF-1, and HDL.

#### Between-group analysis

The between-group analysis found that FMD->Control participants after six FMD cycles displayed a significant (*p* < 0.016) reduction in serum leptin levels, insulin, glucose, total cholesterol, LDL, CRP, AST and ALT, and HOMA-IR and an increase in ghrelin serum levels and HOMA %S with respect to Control->FMD subjects after 6 months on the control diet. A trend for a reduced level was observed for IGF-1 (*p* = 0.029) and ESR (*p* = 0.017), but no significant (*p* > 0.016) differences were found for HDL, TGs, conjugated and unconjugated bilirubin, uremia, serum creatinine, and HOMA %B. FMD->Control participants displayed a significantly (*p* < 0.016) lower WC, weight, BMI, and estimated FM (in both % and kg) and a higher estimated MM (in both % and kg), but only a trend for a lower VF level (*p* = 0.02) (Table S3).

A comparison of Control->FMD participants after cross-over and receiving 6 FMD cycles and FMD->Control participants after a 6-month washout post FMD found LDL, serum glucose, and uremia to be significantly (*p* < 0.016) lower in the Control->FMD arm with respect to the FMD->Control arm. A trend for a reduced level was also observed for IGF-1 (*p* = 0.02), but no significant (*p* > 0.016) differences were observed for HDL, leptin, ghrelin, insulin, total cholesterol, TGs, ESR, CRP, conjugated and unconjugated bilirubin, AST, ALT and serum creatinine, HOMA %B, HOMA %S, HOMA-IR nor in WC, weight, BMI, as well as in estimated FM (in both % and kg), MM (in both % and kg), and VF levels (Table S3).

Weak positive correlations were found between weight loss and Δ serum glucose (R = 0.25, *p* = 0.02) and Δinsulin (R = 0.24, *p* = 0.03), and a weak negative correlation was found between weight loss and Δghrelin (R = −0.23, *p* = 0.03). A moderate and a strong positive correlation was found between weight loss and, respectively, ΔWC (R = 0.4, *p* < 0.001) and ΔBMI (R = 0.93, *p* < 0.001) (Table S1).

#### Safety of FMD, effect on medication, and adherence

The most common self-reported grade 1 (mild) or grade 2 (moderate) symptoms experienced after FMD cycles were weakness, headache, muscle pain, dizziness, and dry mouth. No adverse effects of grade 3 or higher were reported (Figure S1). By means of the provided diaries, at least 80% of participants strictly followed the 5-day FMD prescriptions each month and were considered fully adherent. A minor portion instead partially followed the FMD cycle in one or more days of the cycle (Figure S2).

Five and 1 FMD->Control participants taking antidiabetic medications dropped out of the protocol before T1 and T2, respectively, and 1 and 4 Control->FMD participants taking antidiabetic medications dropped out the protocol before T1 and T2, respectively. Immediately after 6 FMD cycles, drug dosage was reduced in 11 out of 16 (68%) FMD->Control arm participants taking antidiabetic medications and in 10 out of 17 (58%) Control->FMD participants taking antidiabetic medications (Figure 2B). None of the patients increased antihyperglycemic medication after receiving FMD cycles. Three of the 11 patients belonging to FMD->Control who reduced diabetes medication returned to baseline medication prescription after the 6-month washout without FMD. Notably, 4 out of the 21 patients (19%) belonging to the Control->FMD arm increased antihyperglycemic medication during the first 6-month period while only undergoing the control diet (Figure 2B).

Among patients taking antihypertensive mono- or polytherapy, dosage was reduced immediately after the 6 FMD cycles in 4 out of 16 participants (25%) and 5 out of 17 (29%) patients, respectively, belonging to arms 1 and 2. Notably, among those taking antihypertensive medications, 4 FMD->Control and 4 Control->FMD participants dropped out of the trial while receiving FMD cycles, thus not allowing the assessment of the effect of FMD cycles in a larger group. One FMD->Control participant returned to baseline posology in the 6-month washout period whereas 2 Control->FMD participants increased the dosage during the first 6 months on the control diet (Figure 2B).

In line with previous experiences, when evaluating the adherence diaries, no participants pointed to effects of season and/or weekday/weekend days on the effect of the FMD intervention.^17^^,^^18^^,^^21^

## Discussion

This study shows significant—parallel—improvement in taste and smell sensitivity after FMD cycles using both between-group and within-subject analyses, performed by means of both a per-protocol and intention-to-treat analysis (Table 2; Table 3; Table S2; Table S3; Figure 2; Figure 3; Figure 4). OT, OI, TDI, sweet component, and TTS were the main outcome variables found to be significantly improved after the FMD 6-month period but not in the control diet group. Similar results were observed after the control arm group crossed over to receive 6 cycles of the FMD (Table 2). Notably, the portion of patients with hyposmia was reduced by nearly 6-fold with long-lasting effects of FMD cycles on taste and smell sensitivity (Table 2; Figure 2).

Despite its potential for disease prevention and treatment, prolonged water-only fasting is difficult to implement in human subjects and may exacerbate preexisting nutritional deficiencies, making it not feasible and/or safe for children, the elderly, frail individuals, and even most of the healthy adults.^22^ Many studies have shown that an FMD based on animal studies and designed to achieve effects similar to those caused by water-only fasting on IGF-1, insulin-like growth factor-binding protein 1, glucose, and ketone bodies is a more practical and safer dietary intervention than fasting and could have equal or improved effects on markers or risk factors for aging and diseases.^17^^,^^18^^,^^21^^,^^23^^,^^24^ In order to minimize malnourishment and maximize compliance, this approach provided between 3,000 and 4,600 kJ per day, as well as high micronutrient nourishment, to each human subject.^25^ Thus, the wide range of beneficial effects shown here may or may not be superior to those achieved with long-term dietary changes,^12^^,^^26^ but allow subjects to maintain their normal dietary habits in the 25–26 days following the FMD cycle each month. Notably, the many improvements maintained after 6 months from the end of the FMD cycles raise the possibility, which remains to be tested, that FMD cycles could also be partially effective if received only every 3–4 months and could be at least partially effective while rendering this approach even more feasible by requiring a change in dietary habits for only 15–20 days per year.

Further, these results provide strong initial evidence for the ability of FMD cycles to improve taste and smell function while also accomplishing improvements in many risk factors for cardiometabolic disease not simply by causing weight loss. Indeed, only sweet improvement was weakly associated with weight loss and—conversely—sour improvement was weakly associated with a reduction in weight loss (Table S1). Among other marker changes, only ghrelin, serum glucose, insulin, WC, and BMI improvement after the FMD cycles were associated with weight loss, with correlations ranging from weak to strong (see Table S1). These results are consistent with a multi-systemic “reset” caused by FMD cycles that may involve autophagy as well as stem cell activation and/or cellular epigenetic reprogramming, all effects extensively documented in mice treated with FMD cycles but which are also beginning to emerge in other clinical studies.^27^^,^^22^

Previous studies hypothesized that the chemosensory shift could be the consequence of the reduced intake of sweet and energy-dense foods^3^ via a modulation of a mosaic of biochemical regulators, including leptin, ghrelin, insulin, and IGF-1 involved in smell, taste, food intake, body weight, energy expenditure,^2^^,^^28^ and in the appetitive response to food cues by increasing hedonic and incentive responses to adequately influence food intake.^29^ Their receptors—indeed—have been found in the nasal olfactory epithelium, in the olfactory bulb, in the taste buds, and in other brain areas joined with the olfactory system.^28^^,^^30^ An interesting possibility to be tested further is that FMD cycles could also cause reduced inflammation, as shown in animal studies, which could alter peripheral structures and neural pathways involved in chemosensory processing.^25^^,^^31^^,^^32^

An increase in leptin, insulin, and IGF-1 and a reduction in ghrelin were established in previous studies of patients affected by OW and I–II stages of obesity.^7^^,^^9^^,^^17^ FMD cycles partially reversed these effects. Interestingly, ghrelin, leptin, and insulin continued to be significantly different from baseline in FMD->Control even after the 6-month washout period, leading to the hypotheses that many of the effects of the FMD are long lasting.

FMD cycles reduced, in both arms, cardiometabolic markers/risk factors, which were accompanied by a strong reduction in FM but no loss in lean body mass. Although weight loss did not correlate with cardiometabolic improvements, it may have contributed to them.

In agreement with a previous study on diabetic patients,^18^ in the present study, FMD cycles caused a reduction in diabetes and hypertension medications in about 60%–70% and 25%–29%, respectively, of subjects on these medications (Figure 2B). The dropout rate of the present study during the FMD intervention is in line with previous similar experiences^17^^,^^18^ and ranged from 19% to 20%—with a non-compliance rate ranging from 14.8% to 16% in the 2 FMD treatment periods, respectively (Figure 1). This was also due to an overall very good tolerance in terms of adverse effects (Figure S1).^17^^,^^18^

### Limitations of the study

Further tests evaluating perceived preference, liking, pleasantness, and palatability to food-related smell and taste component as well as including retronasal olfactory perception or—for example—fat taste component could have added more data possibly linking smell and taste behavior with FMD impact on eating behavior. We did not establish whether FMD cycles may have promoted long-term lifestyle changes that could have contributed to the improvements reported. However, a similar study applying 12 FMD cycles to treat diabetic subjects concluded that this dietary intervention did not affect diet quality in between FMD periods although it was associated with a small increase in physical activity.^33^ Thus, further and longer studies are necessary to determine whether FMD cycles could promote lifestyle changes that could strengthen the effects of FMD observed in the absence of lifestyle changes.^33^

Since chemosensory tests are different across the literature in terms of reliability and normative values in both healthy and diseased conditions^3^^,^^12^^,^^34^^,^^35^^,^^36^^,^^37^^,^^38^ and because retronasal and orthonasal olfactory testing demonstrated to be equally affected by obesity/OW condition—with some differences in techniques performed across studies^36^^,^^37^—the authors relied on the—more validated—sniffin’ stick test and taste strips.^20^^,^^39^

Although one study in the literature reported 65% of patients with cancer as not compliant with multiple FMD cycles,^40^ probably due to the lack of involvement of dietitians trained in calorie restriction studies or not familiar with fasting-related practices, the present study reported non-adherence to the FMD in only about 15% of participants. This result corroborates previous studies such as that by van den Burg and colleagues on 100 diabetic patients in which 84% of subjects in the FMD arm and 79.6% in the control arm completed the trial.^41^ Only approximately 25% of the FMD group patients did not complete all 12 consecutive cycles of the FMD. Similarly, in another study by Sulaj et al. on diabetic patients, 75% completed the 6 control Mediterranean diet cycles given for only 5 days a month whereas 76% completed all 6 consecutive cycles of the FMD.^18^ In a study involving patients with prostate cancer with metabolic syndrome, 83% completed the 3 consecutive cycles of the FMD.^23^ Notably, the lack of dual-energy X-ray absorptiometry (DEXA) or magnetic resonance imaging (MRI) data is a limitation of our lean body mass assessment data, since bioelectrical impedance analysis (BIA) measurements are likely to have exaggerated the estimated 4 to 7 kg MM gain. Altered estimations of FM and MM by using BIA—instead of DEXA or MRI—has been documented in the literature, possibly due to confounding effects of multiple variables such as gender, age, ethnicity of the population, the BIA device, its equation, BMI, degree of fat-free mass, and hydratation status.^42^^,^^43^^,^^44^^,^^45^ Thus, the remarkable impact of FMD on MM found in the present study could be related to such low concordance between these methods and reinforces the need for DEXA or MRIs to obtain accurate measurements of lean body mass.^45^^,^^46^ In fact, a previous assessment of lean body mass by DEXA showed no changes in absolute lean body mass after 3 FMD cycles.^17^ For future perspectives, further chemosensory assessments and additional—more reliable—body component evaluations could be performed also involving larger cohorts of patients in order to expand present preliminary data.

Furthermore, participants of this study represent a range of glycemic stages. However, we do report specifically on the number of patients with diabetes and on the effect of FMD cycles on glycemic control and drug use (Figure 2B). Notably, cardiometabolic parameters in this study are secondary outcomes that have been measured to depict the effects of FMD cycles on general health. In fact, together with improvements in a wide range of chemosensory parameters, diabetes-related markers (fasting glucose, insulin, and HOMA) underwent significant improvements or a trend for amelioration. Other studies have specifically focused on this aspect and focused on the impact of the FMD on diabetes.^18^^,^^41^ Finally, although authors are aware that the impact of the study could be limited in underserved communities due to the cost of the commercially available 5-day FMD, a 5-day cycle of the diet tested in the trial costs $179 on amazon US (https://www.amazon.com/ProLon-Fasting-Nutrition-Program-Day/dp/B07KXZ9JX1) compared to the average $1,000/month cost for glucagon-like peptide-1 (GLP-1) agonists (https://www.healthline.com/health-news/heres-how-much-more-ozempic-costs-in-the-u-s-compared-to-other-countries#Cost-of-Ozempic-and-similar-drugs-in-the-U.S.-compared-to-other-countries)*.* Notably, the 5-day FMD would eliminate the cost of all foods and drinks that would normally be consumed during a 5-day period. Furthermore, the studies carried out on FMD and diabetes indicate disease regression in 60%–70% of the patients similarly to what was shown in this trial, suggesting that most patients would eventually drastically reduce FMD cycle frequency and some potentially reduce it to only a few per year or even discontinue at least temporarily the use of FMD cycles as indicated by a portion of patients going into remission.^18^^,^^41^

## Resource availability

### Lead contact

Further information and requests for resources and reagents should be directed to and will be fulfilled by the lead contact, Alessandro Micarelli (alessandromicarelli@yahoo.it).

### Materials availability

This study did not generate new unique reagents.

### Data and code availability

All data reported in this paper will be shared by the lead contact upon request. This paper does not report the original code. Any additional information required to reanalyze the data reported in this paper is available from the lead contact upon request.

## Acknowledgments

The authors thank all study participants and staff members of UNITER ONLUS. The clinical trial and data analysis were performed at University of Rome “Tor Vergata,” UNITER ONLUS, and Institute of Clinical Physiology - National Research Council.

## Author contributions

Conceptualization, A.M., M.A., and V.D.L.; methodology, A.M., S.M.-S., S.M., V.D.L., A.V., I.G., S.C., I.M., I.I., B.M., and V.C.; formal analysis, A.M., S.M.-S., I.G., M.A., V.C., A.V., and S.M.; data curation, A.M., M.A., S.M.-S., S.M., I.G., I.M., I.I., and A.V.; writing – original draft preparation, A.M., V.D.L., and M.A.; writing – review and editing, V.D.L., M.A., A.M., S.M.-S., S.M., A.V., B.M., S.C., and V.C.; supervision, A.M., M.A.,V.D.L., S.M.-S., S.C., S.M., and A.V. All authors have read and agreed to the published version of the manuscript. All the clinical work and data analysis were carried out at University of Rome Tor Vergata and UNITER ONLUS, Rome, Italy.

## Declaration of interests

This research received no external funding. The Prolon 5-day fasting-mimicking diet used in this study was provided by L-Nutra, which had no influence on design and conduct of this study, collection, analysis, and interpretation of the data or on the preparation, review, or approval of this article.

V.D.L. has equity interest in and serves as an advisor of L-Nutra, a company making medical food. He also has filed patents related to fasting-mimicking diets and their medical use. V.D.L., A.M., and M.A have filed a patent on Fasting Mimicking Diet to Improve Chemosensory Function. All of the clinical work was carried out at University of Rome Tor Vergata and UNITER ONLUS, Rome, Italy.

## STAR★Methods

### Key resources table

**Table:** 

REAGENT or RESOURCE	SOURCE	IDENTIFIER
**Biological samples**		

Plasma	Antecubital Venous draw	N/A

**Chemicals, peptides, and recombinant proteins**		

Leptin - immunoassay kit	FineTest, Wuhan, China	cat. No. EH0216
Ghrelin - immunoassay kit	FineTest, Wuhan, China	cat. No. EH0355
Insulin-like growth factor 1 (IGF-1) - immunoassay kit	FineTest, Wuhan, China	cat. No. EH0165
Insulin - immunochemistry assay	Cobas® e801	Roche Diagnostics Italia S.p.a., Italy
Serum glucose	Alinity C	Abbott Laboratories, Illinois, USA
Total cholesterol	Alinity C	Abbott Laboratories, Illinois, USA
Low-density lipoprotein cholesterol - LDL	Alinity C	Abbott Laboratories, Illinois, USA
High-density lipoprotein cholesterol - HDL	Alinity C	Abbott Laboratories, Illinois, USA
Triglycerides - TGs	Alinity C	Abbott Laboratories, Illinois, USA
Conjugated bilirubin	Alinity C	Abbott Laboratories, Illinois, USA
Unconjugated bilirubin	Alinity C	Abbott Laboratories, Illinois, USA
Erythrocyte sedimentation rate - ESR	TEST1 2.0	Alifax S.r.l., Padua, Italy
C-reactive protein - CRP	Alinity C	Abbott Laboratories, Illinois, USA
Aspartate aminotransferase - AST	Alinity C	Abbott Laboratories, Illinois, USA
Alanine aminotransferase - ALT	Alinity C	Abbott Laboratories, Illinois, USA
Uraemia	Alinity C	Abbott Laboratories, Illinois, USA
Serum creatinine	Alinity C	Abbott Laboratories, Illinois, USA

**Software and algorithms**		

STATISTICA 7 package for Windows	StatSoft Inc., Oklahoma, USA	N/A
GraphPad Prism version 6.00 for Windows	GraphPad Software, California, USA	N/A
https://www.dtu.ox.ac.uk/homacalculator/index.php	https://www.dtu.ox.ac.uk/homacalculator/index.php	N/A

**Other**		

Sniffin’ Stick tests	Burghart Instruments, Wedel, Germany	Cat#200113
Taste Strips	Burghart Instruments, Wedel, Germany	Cat# 200114
Bioelectrical impedance analysis device	Omron HBF-500 BIA, Omron Medizintechnik, Mannheim, Germany	N/A
Scale	Seca model 700, Seca GmbH, Hamburg, Germany	N/A
Stadiometer	Holtain Ltd, UK	N/A

### Experimental model and study participants details

#### Participants, study design, exclusion/inclusion criteria

One hundred thirteen Caucasian adults participants with BMI ≥25 were were recruited from the University Hospital of Rome ‘Tor Vergata’. Recruitment of subjects was based on fliers, institutional website, and/or word of mouth. All the participants completed and signed a written informed consent. The study was performed in agreement of the Declaration of Helsinki and was approved by the Institutional Ethics Committee (Reference number RS 60/20, date of vote: 2020-7-24; ClinicalTrials.gov; NCT04529161). Flow of participant enrollment and participation was prepared following the CONSORT standards for randomized clinical trials with crossover design. Inclusion criteria were BMI ≥25 and 18 to 75 years of age. All the participants underwent a general clinical and ear-nose-throat (ENT) examination. Chemosensory perception disturbances related to previous COVID-19 infection were considered as pre-enrollment exclusion criteria. Current or recent smokers (<3 years of abstinence) and individuals affected by allergies and history of ENT surgery were excluded. Legally incapacitated persons were excluded; individuals suffering from major systemic or organ failure disorders including neurodegenerative, psychiatric and cardiovascular disorders, nondiabetic liver disease, diabetes mellitus type 1, pancreatogenic diabetes, or steroid-induced diabetes, as evaluated by medical history, physical and neuropsychological examination and routine blood tests were further excluded. Acute infection/fever, history of cancer disease in the last 5 years prior to study, infectious hepatitis B, C, or E, HIV infection, autoimmune diseases or immunosuppressive therapy, participation in other interventional studies; anemia or hematological disease, polyneuropathy (autoimmune, alcohol-induced, or vitamin B12 defciency, collagenosis), pacemaker and food allergy (nuts, tomato, soja, or other ingredients enlisted in the diet program) were considered as exclusion criteria. Conditions of vegetarian/vegan diet, ongoing use of medication possibly impacting chemosensory perception and drugs/alcohol abuse were considered as exclusion criteria. Gastrointestinal/eating disturbances and surgery (also detection of Helicobacter pylori excluded by a C13 urea breath test, but not history of appendectomy) and history of gustatory and/or smelling disorders were considered as further drop out conditions. Pregnant and currently breastfeeding females were excluded.^4^^,^^9^ Participants suffering from anosmia (i.e., TDI ≤16.5) were excluded.^39^ Participants demonstrating ageusia when tested according to previous procedures were excluded.^20^

#### Randomisation and masking

Because this was a dietary intervention study, it was not possible for participants or all study personnel to be blinded to group assignment. However, study personnel involved in participants enrollment, data collection and specimen analysis were blinded to group assignments.

Eligible participants were randomly assigned to either FMD->Control (participants following FMD diet in the first semester) or Control->FMD (participants following FMD diet in the second semester) groups of the study by using a stratified computed procedure for gender, age (cutoff value 54 years) and BMI (cutoff value 30 kg/m^2^) generated by an external statistician. The randomisation was open for participants and research staff, but outcome assessors were masked during statistical analyses.

After having received one individual dietary counseling before the baseline visit, FMD->Control and Control->FMD participants were instructed to comply for 5 consecutive days each month with FMD for the first and second 6-months period, respectively. During the second and the first 6-months period, respectively, FMD->Control and Control->FMD participants were instructed to maintain their regular eating habits. During the FMD 6-months period they were further instructed to return to their normal diet after completion until the next cycle that was initiated about 25 days later.

#### Supplementary results—participants

During the first semester 10 (mean age = 49.2 ± 8.5 years; 4 females; BMI = 35.03 ± 4.88 kg/m^2^) and 5 (mean age = 55.6 ± 9.5 years; 2 females; BMI = 34.04 ± 6.97 kg/m^2^) participants respectively belonging to FMD->Control and Control->FMD dropped out. During the second semester, 3 (mean age = 45 ± 7.54 years; 1 female; BMI = 31.4 ± 2.33 kg/m^2^) and 9 (mean age = 41 ± 14.17 years; 6 females; BMI = 31.96 ± 3.29 kg/m^2^) participants respectively belonging to FMD->Control and Control->FMD dropped out the study. A total of 78 patients (40 FMD->Control and 38 Control->FMD) completed 6 consecutive FMD cycles (Figure 1).

When considering only the participants who completed the FMD semester, hyposmia was found in 32.5% (13/40) and 10% (4/40) FMD->Control (5 hyposmic patients dropped out before the end of the FMD semester) and in 34.2% (13/38) and 2.6% (1/38) Control->FMD (6 hyposmic patients dropped out before the end of the FMD semester) patients at T0 and T1, respectively. Thus, the total number of hyposmic FMD patients decreased by 33.3% (26/78) to 6.4% (5/78), a 5.2-fold decrease (Figure 2).

When comparing main baseline parameters of participants with worsened TDI (*n* = 5; 3 males, 2 females, ΔTDI = −2.6 ± 0.54 in participants undergoing FMD and n) with those who improved in TDI (*n* = 73; 34 males, 39 females; ΔTDI = 3.51 ± 1.89) no significant differences were found in terms of weight (97.24 ± 17.9 kg vs. 95.66 ± 13.82 kg, *p*-value = 0.8), BMI (34.06 ± 4.23 kg/m^2^ vs. 33.82 ± 3.85 kg/m^2^, *p*-value = 0.89), WC (113.38 ± 7.5 cm vs. 112.34 ± 9.2 cm, *p*-value = 0.8) and age (62.6 ± 9.91 years vs. 54.23 ± 12.27 years, *p*-value = 0.14). When comparing main baseline parameters of control participants who worsened in TDI (*n* = 29, 11 males, 18 females, ΔTDI = −1.2 ± 1.03) with those who improved in TDI (*n* = 18; 9 males, 9 females; ΔTDI = 0.86 ± 0.96) significant differences were found in terms of weight (91.6 ± 10.91 kg vs. 105.5 ± 14.95 kg, *p*-value <0.001), BMI (32.61 ± 3.29 kg/m^2^ vs. 35.84 ± 3.12 kg/m^2^, *p*-value = 0.001) and WC (109.91 ± 9.96 cm vs. 116.76 ± 6.45 cm, *p*-value = 0.01) but not in terms of age (49.68 ± 14.65 years vs. 55.66 ± 6.56 years, *p*-value = 0.11). When comparing main baseline parameters of participants undergoing FMD with worsened TTS (*n* = 9; 3 males, 6 females, ΔTTS = −3.55 ± 2.54 with those who improved in TTS (*n* = 69; 34 males, 35 females; ΔTTS = 3.84 ± 1.59) no significant differences were found in terms of weight (88.85 ± 15.48 kg vs. 96.67 ± 13.64 kg, *p*-value = 0.11), BMI (34.04 ± 3.98 kg/m^2^ vs. 33.81 ± 3.86 kg/m^2^, *p*-value = 0.87), WC (110.96 ± 8.28 cm vs. 112.60 ± 9.27 cm, *p*-value = 0.61) and age (56.11 ± 12.75 years vs. 54.59 ± 12.27 years, *p*-value = 0.72). Similarly, when comparing main baseline parameters of control participants who worsened in TTS (*n* = 18, 9 males, 9 females, ΔTTS = −1.5 ± 0.92) with those who improved in TTS (*n* = 29; 11 males, 18 females; ΔTTS = 0.82 ± 1.25) no significant differences were found in terms of weight (95.90 ± 14.19 kg vs. 97.58 ± 14.44 kg, *p*-value = 0.69), BMI (32.8 ± 3.71 kg/m^2^ vs. 34.49 ± 3.38 kg/m^2^, *p*-value = 0.11), WC (110.94 ± 11.28 cm vs. 113.83 ± 7.82 cm, *p*-value = 0.23) and age (49.66 ± 12.9 years vs. 53.41 ± 12.18 years, *p*-value = 0.32).

Dropped FMD->Control participants were – although not significantly – older, gender-unbalanced and with higher BMI with respect dropped Control->FMD participants during the respective FMD semester and younger and with lower BMI with respect Control->FMD participants during the respective control semester. Additional significant and not significant differences were found in dropped participants in chemosensory testing and main biochemical regulators (Table S4). Finally, 37 FMD->Control (74% of enrolled patients; mean age = 56.5 ± 12.85 years; 18 females; BMI = 33.34 ± 4.38 kg/m^2^) and 38 Control->FMD (73%; of enrolled patients; mean age = 53.7 ± 11.69 years; 21 females; BMI = 34.36 ± 3.48 kg/m^2^) participants completed the study protocol including both the 6 monthly FMD cycles and the control diet periods.

### Method details

#### Diet intervention

The FMD is a plant-based diet designed to attain fasting-like reduction in serum glucose and IGF-1, and increase in IGFBP-1 and ketone bodies while providing both macro- and micronutrients to minimize the burden of fasting and adverse effects.^17^ Day 1 of the FMD supplies ∼4600 kJ (11% protein, 46% fat, and 43% carbohydrate), whereas days 2–5 provide ∼3000 kJ (9% protein, 44% fat, and 47% carbohydrate) per day. The FMD comprises proprietary formulations belonging to the University of Southern California and licensed to L-Nutra (www.prolonfmd.com) of vegetable-based soups, energy bars, energy drinks, chip snacks, tea, and a supplement providing high levels of minerals, vitamins, and essential fatty acids. All items to be consumed per day were individually boxed to allow the subjects to choose when to eat while avoiding accidentally consuming components of the following day and reducing the likelihood of other sources of intake rather than the boxed ones. The first cycle of the FMD started on the first feasible day after the baseline visit. All the participants were followed by a physician, a nutritionist and a neuropsychologist by means of 24-h telephone platform, instant messaging and on-call visits. Oral antidiabetic therapy was discontinued during FMD.^18^ Antihypertensive medication was reduced in case of hypotension (lower than 100 mmHg for systolic and lower than 60 mmHg for diastolic values). All participants were instructed to avoid excessive physical activity during FMD and to return to their normal physical activity afterward.^18^ Adherence and the possible impact of week and weekend days were monitored by means of diaries provided at the beginning of the study to each participant in the intervention group to record their consumption of the test product. Participants were also asked to document any additional food or beverage items consumed during the five-day period beyond the provided meal kit.^18^^,^^21^

#### Safety and COVID-19 pandemic preventive measures

Study participants were asked about adverse events at each study visit; events were graded according to the general Common Terminology Criteria for Adverse Events (CTCAE) (v4.0) guidelines (see the Supplementary Materials for details). Considering the nature of the chemosensory testing which unavoidably are conducted with close contact between investigator and patient, and to protect both, preventive measures including a nasal swab test before and after the visit, face mask and gloves clothing, a script for COVID-19 related symptoms, temperature monitoring before the visit as well as hand and surface sanitizer were implemented. No guests were allowed during the study visit which duration was limited as much as possible and the rooms were decontaminated after each visit.^47^

Participants completed baseline (T0) and follow-up examinations at the end of the first (T1) and second 6-months period (T2). In the FMD 6-months period examinations were performed after a washout period of 5–7 days of normal caloric intake after the sixth FMD cycle. After a 12-h fast, between 7:00 to 9:30 a.m.^48^ all the participants underwent at each time-point.

### Primary outcomes

#### Chemosensory testing

*Olfactory function testing*: smell function was assessed by means of the commercially available Sniffin’ Sticks test battery (Sniffin’ Sticks; Burghart Instruments, Wedel, Germany), a well-recognized tool to evaluate olfactory performance in clinical and research context.^49^ It includes subtests for odor threshold (OT), odor discrimination (OD) and odor identification (OI), which are associated with different aspects of olfactory processing along the neural stream from olfactory bulb to the olfactory cortex^50^ and were operationalized following previous procedures.^4^^,^^49^ An interval of 3–5 min was applied between each subtest.^51^ In each test the sum of correct answers can range from 0 to 16. The sum of all three subtests results in the composite TDI-score and reflects general olfactory capacity which thus can range from 0 to 48, with higher scores depicting a greater funcionality.^39^ To adjust for well-known gender differences in olfactory abilities and the age range of our cohort,^52^ we then categorized olfactory performance as abnormal using the <25th percentile TDI value from age and gender-adjusted normative data (cut-off values for females were 32.35, 33.5, 33.5, 32.5, 30.75, 29.13 and 25.5 and for male were 30.75, 32.75, 32.76, 30.44, 29.25, 28.5, 22.75, respectively for 11–20, 21–30, 31–40, 41–50, 51–60, 61–70 and 71–80 years sub-groups).^53^

*Taste function testing:* the taste test - consisting of filter paper strips (“Taste Strips”, Burghart Instruments, Wedel, Germany) impregnated with four concentrations of the four basic taste qualities: sweet, sour, salty and bitter (for details see^54^) - is a semi-quantitative, accurate, quick and easy tool to only investigate the threshold of both side of the tongue for each of the four basic tastants which are administered in increasing concentrations (0.05, 0.1, 0.2, 0.4 g/mL sucrose; sour: 0.05, 0.09, 0.165, 0.3 g/mL citric acid; salty: 0.016, 0.04, 0.1, 0.25 g/mL sodium chloride; bitter: 0.0004, 0.0009, 0.0024, 0.006 g/mL quinine hydrochloride) and by means of a randomized method on the left or right side of the anterior third of the extended tongue, resulting in a total of 32 trials. Before each administration of a strip, the mouth was rinsed with water. With their tongue still extended, participants were asked – by means of a multiple forced choice method - to identify the taste from a list of the four qualities. After the number of correctly identified tastes per side was summed, the left and right sides scores were added up in order to obtain total number of identified tastant (TTS) that can range from 0 to 32, with higher scores depicting a greater funcionality.^54^ The procedure lasted about 20 min for the lateralized testing.^20^ To adjust for well-known gender differences in gustatory abilities and the age range of our cohort,^20^ we then categorized taste performance as abnormal using the <10th percentile TTS value from age and gender-adjusted normative data (cut-off values for female were 19, 15 and 10.2 and for male were 17, 9 and 9 respectively for 18–40, 41–60 and >60 years sub-groups).^20^

### Secondary outcomes

#### Biochemical assays and anthropometric measures

Baseline laboratory parameters, including serum glucose, alanine aminotransferase (ALT) and aspartate aminotransferase (AST), total cholesterol, triglycerides (TGs), high-density lipoprotein (HDL) cholesterol and low-density lipoprotein (LDL) cholesterol, C-reactive protein (CRP), erythrocyte sedimentation rate (ESR), conjugated and unconjugated bilirubin, uraemia and serum creatinine were measured under standardized condition in the institutional laboratory (Alinity C, Abbott Laboratories, Illinois, USA and TEST1 2.0, Alifax S.r.l., Padua, Italy). Five mL of blood were drawn from the antecubital vein in heparinized vacuum tubes. Samples collected were centrifuged for 5 min at 3000 x g to separate plasma. All plasma samples were stored in multiple aliquots, immediately frozen at −80°C, until assayed within one month from the collection.^9^ Insulin was analyzed by a Cobas e801 (Roche Diagnostics Italia S.p.a., Monza (MB), Italy) analytical unit which is a high throughput immunochemistry module. Leptin levels were measured by means of enzyme immunoassay (ELISA) kit (cat. No. EH0216; FineTest, Wuhan, China), ghrelin and IGF-1 levels by an enzyme linked immunosorbent assay kit respectively (cat. No. EH0355; FineTest, Wuhan, China) and (cat. No. EH0165; FineTest, Wuhan, China). These latter analysis were carried out in accordance with the manufacturer’s instructions, and the concentrations were measured spectrophotometrically at a wavelength of 450 nm by comparing the samples’ optical density to standard curves. All the samples and standards were read by a microplate reader spectrophotometer (Infinite M200, Tecan Group Ltd., Männedorf, Switzerland).^9^ The Homeostasis Model Assessment of insulin resistance (HOMA-IR), steady state beta cell function (%B) and insulin sensitivity (%S), were calculated from fasting insulin and glucose by means of the HOMA2 Calculator (https://www.dtu.ox.ac.uk/homacalculator/index.php).^55^^,^^56^

#### Anthropometric measures

Height and body weight were measured twice by the same examiner throughout the study with a scale (Seca model 700; Seca GmbH, Hamburg, Germany) and stadiometer (Holtain Ltd, UK) to the nearest 0.1 kg and 0.01 m, respectively.^57^^,^^58^ During the measurements, subjects wore only underwear. BMI was calculated by dividing the body weight (Kg) by height in meters and was expressed as kg/m^2^. Waist circumference (WC) were measured twice in a standing position with a non-elastic tape measure, while participants were instructed to breathe out mildly, at the midpoint between the top of the iliac crest and the lowest coastal rib.^59^ Following previous experiences and bioelectrical impedance analysis (BIA) devices (Omron HBF-500 BIA, Omron Medizintechnik, Mannheim, Germany) an estimation of fat mass (FM, in % and Kg), skeletal muscle mass (MM, in % and Kg) and grade of visceral fat (VF level) was calculated by means of the manufacturers’ equations.^60^^,^^61^

### Quantification and statistical analysis

#### Data handling and statistical analysis

A total sample size of at least 24 and 62 subjects was estimated to detect a mean difference between groups of 20% (6.8) and 16% (4.12) reduction in mean TDI and TTS respectively, with a two-sample t-test and a Bonferroni-adjusted two-sided significance level of α = 0.025 and a power of at least 80%. The estimated control group mean (SD) TDI of 34 (5.5) and TTS of 25.8 (5.3) used gender- and age-balanced published data.^7^^,^^9^^,^^39^ The choice to use the TDI and TTS composite score – rather than their sub-items – as main outcome measures is supported by the literature^2^^,^^4^^,^^7^^,^^9^^,^^54^ and it is related to their main clinical significance in diagnosis and follow-up of olfactory and taste performance.^20^^,^^39^ The sample was further enlarged due to an estimated dropout associated with FMD intervention (15%) and due to COVID-19 outbreak which - burdening the olfactory and gustatory perception of patients – could have further dropped patients out of the study. The X^2^ test was performed to assess associations between categorical factors and groups. Descriptive data are shown as mean ± SD for normally distributed variables, median (25^th^ [Q1], 75^th^ [Q3] percentile, IQR) for log-normally distributed variables, and frequencies for categorical variables. Distribution assumption was assessed visually and evaluated by the Kolmogorov-Smirnov test. Primary comparison using paired two-tailed Student’s t tests involved changes from baseline within the treatment arms (FMD->Control; *n* = 40 and Control->FMD; *n* = 38) and in participants following their regular eating habits in the first semester (Control->FMD; *n* = 47) who served as control diet group and *p* value <0.01 was considered significant. A further observational analysis between the combination of pre-post FMD differences (Δ) in participants following FMD in the first semester (FMD->Control; *n* = 40) and in the second semester (Control->FMD; *n* = 38) and participants following 6-month control diet (Control->FMD; *n* = 47) was performed using two-tailed two-sample t-tests, and *p* value <0.01 was considered significant. Analyses were performed by means of both a per protocol and an intention-to-treat approach. Post-hoc comparison between main baseline parameters in participants not improving in terms of TDI and TTS was achieved by means of two-tailed two-sample t-tests with respect participants in which improved chemosensory perception scores were found after having completed the FMD 6-month or the control period and *p* value <0.01 was considered significant. A between-group analysis of variance was carried out for each taste and smell testing score as well as biochemical assay and anthropometric variables at T0, T1 and T2 in all the participants. Gender and age were treated as categorical and continuous predictors, respectively and time points and arms were used as factors for the main outcome measures. The significant cut-off level (α) was set at a *p* value of 0.05. Bonferroni correction for multiple comparisons was used for the post hoc test of the significant main effects, and the corrected level of significance was set at 0.016 (0.05/3). In this case analyses were performed in the intention-to-treat population. Finally, a two-tailed Spearman’s rank correlation was performed between Δ chemosensory testing, biochemical assays and anthropometric measures variables and Δ weight, considering FMD patients values as ‘a continuum’.^62^ A significant cut-off level (α) was set at a *p* value of 0.05 and the magnitude was defined as negligible, weak, moderate, strong or very strong respectively for R = 0–0.09, R = 0.1–0.39, R = 0.4–0.69, R = 0.7–0.89 and R = 0.9–1.^63^ (STATISTICA 7 package for Windows, Statsoft Inc., Oklahoma, USA and GraphPad Prism version 6.00 for Windows, GraphPad Software, California, USA for visualization).

### Additional resources

The trial is registered at ClinicalTrials.gov (NCT04529161; https://clinicaltrials.gov/study/NCT04529161?tab=table).

## Supplemental information

Document S1. Figures S1 and S2 and Tables S1, S3, and S4

Table S2. Changes in biochemical assays and anthropometric measures by means of per-protocol and intention-to-treat analysis, related to Table 2

Document S2. Article plus supplemental information
