# Supplementary material for: Chemosensory and cardiometabolic improvements after a fasting-mimicking diet: A randomized cross-over clinical trial
Source: Cell Rep Med. 2025 Feb 18;6(2):101971. doi: 10.1016/j.xcrm.2025.101971 (PMC11866515; doi:10.1016/j.xcrm.2025.101971)
Supplement: Document S1. Figures S1 and S2 and Tables S1, S3, and S4 [file mmc1.pdf]

Cell Reports Medicine, Volume *6*

Supplemental information

Chemosensory and cardiometabolic improvements after a fasting-mimicking diet: A randomized cross-over clinical trial

Alessandro Micarelli, Simona Mrakic-Sposta, Alessandra Vezzoli, Sandro Malacrida, Sara Caputo, Beatrice Micarelli, Ilaria Misici, Valentina Carbini, Ilaria Iennaco, Ivan Granito, Valter D. Longo, and Marco Alessandrini

Supplementary Figures

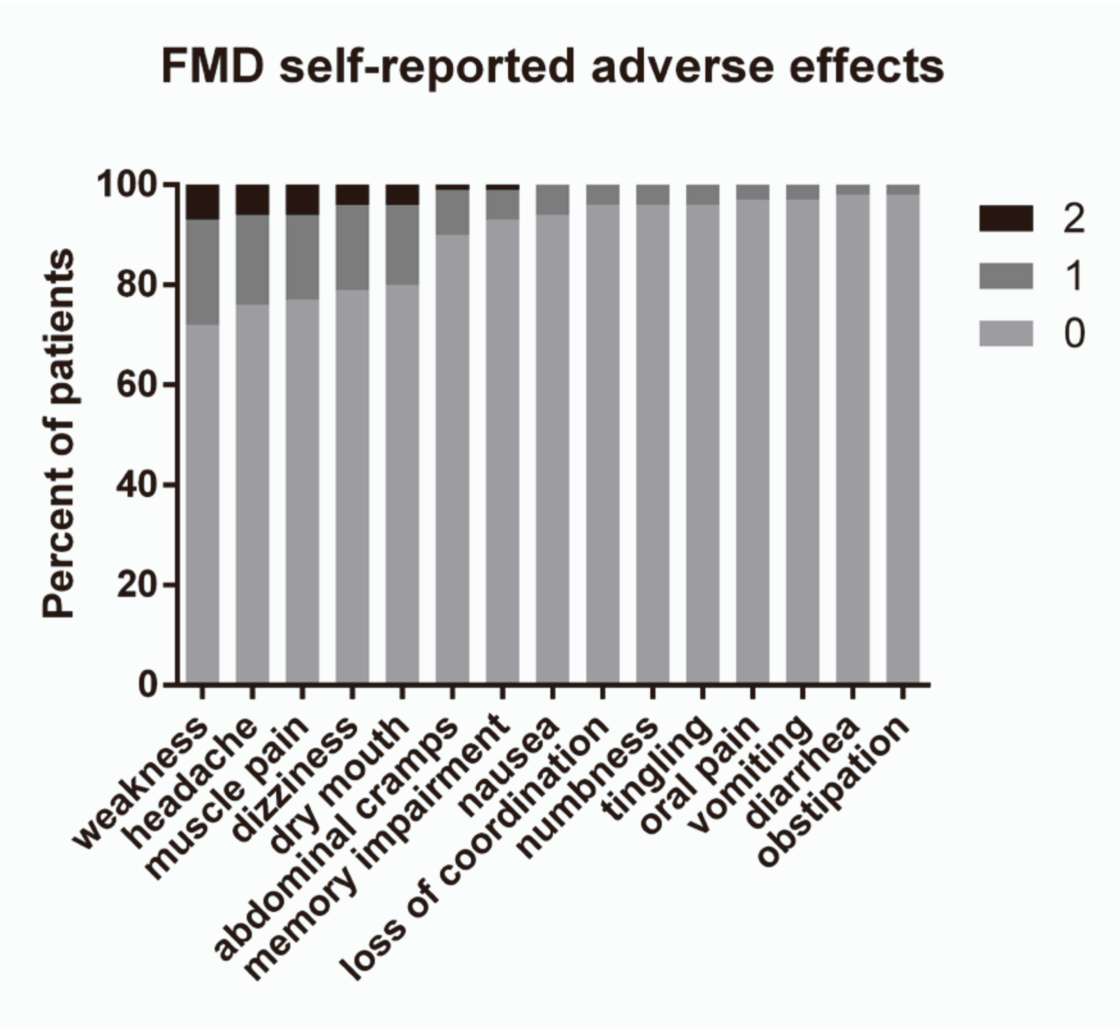

Figure S1. Percentage of participants reporting self-reported adverse effects based on Common Terminology Criteria for Adverse Events: no adverse effect (grade 0), grade 1 (mild), or grade 2 (moderate) adverse effects; grades 3 (severe), 4 (life-threatening) and 5 (death) were not reported. Related to "STAR Methods - Safety and COVID-19 pandemic preventive measures".

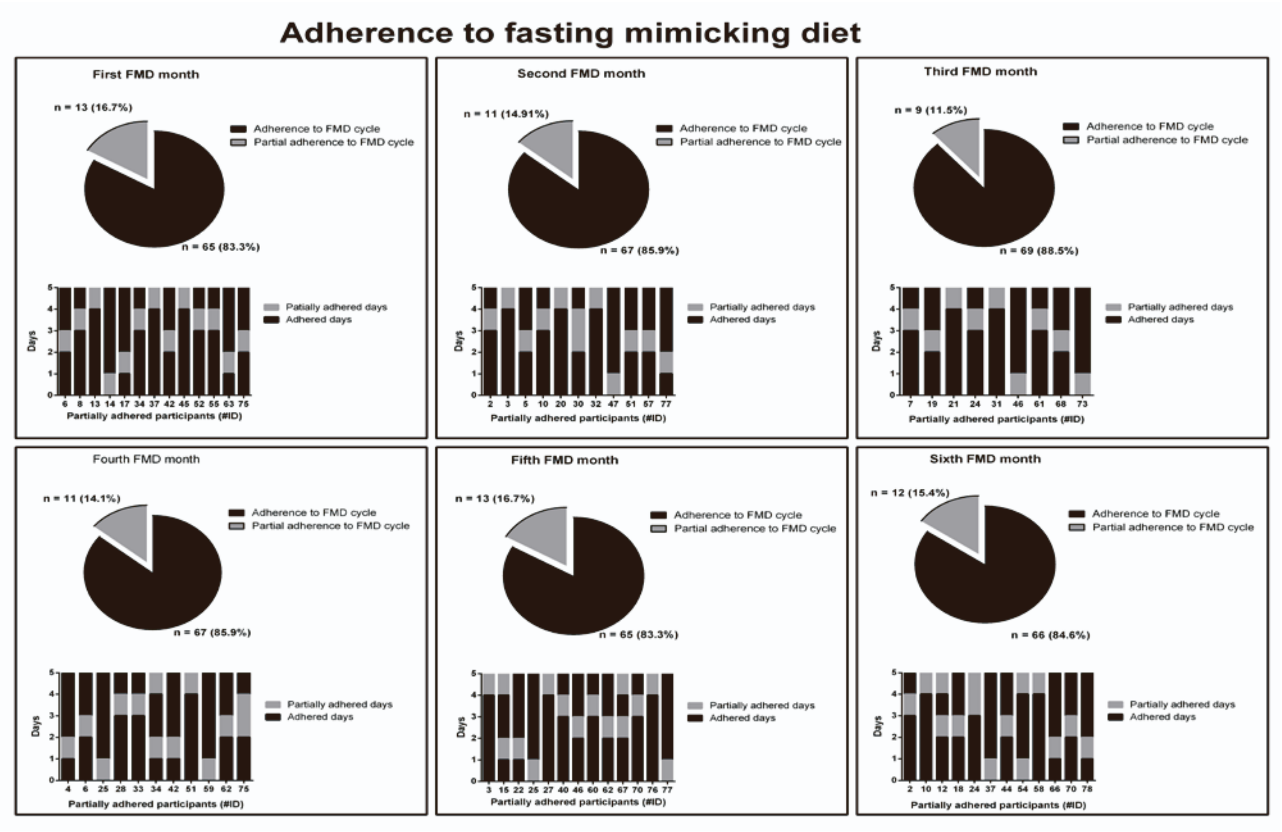

Figure S2. Pie charts reporting number (and percentage) of participants (n = 78) who completed each fasting mimicking diet (FMD) cycle fully or partially adhering to each day FMD prescription. In histograms single day full or partial adherence of monthly FMD cycle is reported for each participant (identified with #ID). Related to “STAR Methods - Diet Intervention”

Supplementary Tables

Table S1. Correlation between weight loss and changes in chemosensory testing, biochemical assays and anthropometric measures in participants completing the trial. Related to "STAR Methods - Data handling and statistical analysis".

	**Difference: Mean ± SD**	**Δ Weight correlation (Spearman)**	**p**
ΔOT	1.45 ± 1.08	r = - 0.19	0.08
ΔOD	1.06 ± 1.2	r = 0.07	0.51
ΔOI	0.51 ± 0.92	r = 0.19	0.08
ΔTDI	3.11 ± 2.37	r = - 0.04	0.7
ΔSweet	0.91 ± 0.98	**r = - 0.23**	**0.03**
ΔSour	0.88 ± 0.95	**r = 0.22**	**0.04**
ΔSalty	0.52 ± 1.07	r = - 0.1	0.38
ΔBitter	0.8 ± 1.05	r = - 0.08	0.48
ΔTTS	2.98 ± 2.92	r = - 0.21	0.06
ΔLeptin (ng/ml)	-8.88 ± 5.38	r = 0.02	0.81
ΔGhrelin (pg/ml)	47.2 ± 35.67	**r = - 0.23**	**0.03**
ΔIGF-1 (ng/ml)	-16.93 ± 20.31	r = 0.14	0.21

ΔSerum glucose (mg/dl)	-10.26 ± 8.4	**r = 0.25**	**0.02**
ΔInsulin (µU/ml)	-4.26 ± 3.88	**r = 0.24**	**0.03**
ΔTotal cholesterol (mg/dl)	-31.75 ± 20.34	r = - 0.02	0.81
ΔLDL (mg/dl)	-27.38 ± 19.27	r = 0.06	0.58
ΔHDL (mg/dl)	3.85 ± 11.69	r = - 0.05	0.64
ΔTGs (mg/dl)	-24.62 ± 29.55	r = 0.15	0.18
ΔConjugated bilirubin (mg/dl)	-0.01 ± 0.09	r = - 0.13	0.23
ΔUnconjugated bilirubin (mg/dl)	-0.01 ± 0.15	r = - 0.03	0.76
ΔESR (mm/h)	-4.02 ± 4.31	r = - 0.01	0.88
ΔCRP (mg/L)	-0.73 ± 1.64	r = 0.09	0.4
ΔAST (U/L)	-4.67 ± 5.37	r = 0.19	0.08
ΔALT (U/L)	-7.32 ± 8.97	r = 0.17	0.11
ΔUraemia (mg/dl)	-4.66 ± 7.78	r = - 0.06	0.58
ΔSerum creatinine (mg/dl)	-0.04 ± 0.09	r = 0.04	0.68
HOMA %B	-6.23 ± 24.56	r = 0.07	0.52
HOMA %S	36.04 ± 32.08	r = 0.002	0.98

HOMA IR	-5.24 ± 24.08	r = 0.16	0.13
ΔWC (cm)	-7.03 ± 4.53	**r = 0.4**	**< 0.001**
ΔBMI (Kg/m^2)	-2.33 ± 1.1	**r = 0.93**	**< 0.001**
ΔFM%*	-9.56 ± 8.17	r = 0.12	0.26
ΔFM (Kg)*	-10.47 ± 8.45	r = 0.21	0.055
ΔMM%*	8.52 ± 8.48	r = - 0.07	0.49
ΔMM (Kg)*	6.2 ± 7.9	r = 0.06	0.58
ΔVFlevel*	-3.02 ± 1.77	r = 0.18	0.1

Table S1. Correlation between weight loss and changes in chemosensory testing, biochemical assays and anthropometric measures in participants completing the trial (n = 78). OT, odor threshold; OD, odor discrimination; OI, odor identification; and their sum (TDI); TTS, total taste score; ALT, alanine aminotransferase; AST, aspartate aminotransferase; TGs, triglycerides; HDL, high density lipoprotein cholesterol; LDL, low-density lipoprotein cholesterol; ESR, erythrocyte sedimentation rate; CRP, C-reactive protein; HOMA %B, steady state beta cell function; HOMA %S, insulin sensitivity; HOMA-IR, homeostasis model assessment of insulin resistance; WC, waist circumference; BMI, body mass index; ng, nanogram; pg, picogram; ml, milliliter; mg, milligram; dl, deciliter; U, international unit; µU, micro international unit; mm, millimiter; L, liter; h, hour; cm, centimeter; m, meter; Kg, kilogram; %, percentage; FM, fat mass; MM, muscle mass; VF, visceral fat. *, estimated by means of bioelectrical impedance analysis. Values are given in mean ± standard deviation (SD). In bold r significant values ($p < 0.05$).

Table S3. Between-group comparisons in biochemical assays and anthropometric measures in all participants at T0, T1 and T2. Related to Table 3.

	T0		p	T1		p	T2		p
	FMD->Control (n = 50)	*Control->FMD (n = 52)*		FMD->Control (n = 40)	Control->FMD (n = 47)		FMD->Control (n = 37)	Control->FMD (n = 38)	
	Mean ± SD (CI)/median (IQR)	Mean ± SD (CI)/median (IQR)		Mean ± SD (CI)/median (IQR)	Mean ± SD (CI)/median (IQR)		Mean ± SD (CI)/median (IQR)	Mean ± SD (CI)/median (IQR)	
Biochemical Assays									
**Leptin (ng/ml)**	26.89 (7.86)	26.31 (14.74)	0.28	18.19 (12.3)	28.56 (17.63)	< 0.001	18.44 (12.78)	21.38 (8.44)	0.23
**Ghrelin (pg/ml)**	197.13 ± 42.13 (185.45 208.8)	203.86 ± 37.74 (193.6 - 214.12)	0.39	251.9 ± 52.31 (235.69 - 268.12)	207.28 ± 37.82 (196.47 - 218.09)	< 0.001	232.51 ± 55.67 (214.57 -250.45)	250.83 ± 50.28 (234.84 -266.82)	0.13
**IGF-1 (ng/ml)**	159.51 ± 23.75 (152.92 -166.09)	157.06 ± 26.38 (149.89 - 164.23)	0.62	144.46 ± 27.01 (136.09 - 152.83)	156.82 ± 24.87 (149.7 - 163.93)	0.029	155.54 ± 32.12 (145.19 -165.89)	140.78 ± 23.7 (133.24 -148.32)	0.02
**Serum glucose (mg/dl)**	99 (13.75)	99 (22.25)	0.59	90 (12)	98 (21.25)	0.007	95 (18)	87 (14.75)	0.01
**Insulin (µU/ml)**	11.49 (7.51)	10.61 (7.1)	0.25	7.95 (5.4)	11.41 (6.43)	< 0.001	9.6 (5.8)	8.96 (6.01)	0.48
**Total cholesterol (mg/dl)**	223.28 ± 38.36 (212.64 - 233.91)	219.59 ± 36.3 (209.72 - 229.46)	0.61	195.6 ± 31.19 (185.93 - 205.26)	217.51 ± 33.44 (207.94 - 227.07)	0.002	195.13 ± 34.23 (184.1 -206.16)	182.15 ± 30.09 (172.58 -191.72)	0.08
**LDL (mg/dl)**	142.32 ± 37.93(131.8 - 152.83)	140.78 ± 35.27 (131.2 - 150.37)	0.83	123.05 ± 24.48 (115.46 - 130.63)	138.93 ± 33.33 (129.4 - 148.46)	0.014	127.64 ± 29.14 (118.25 - 137.03)	100.21 ± 29.78 (90.74 -109.68)	< 0.001
**HDL (mg/dl)**	55 (22.759	52 (19.25)	0.4	57 (15.25)	52 (20)	0.2	55 (11)	60 (15)	0.02
**TGs (mg/dl)**	111 (76.25)	115 (41.25)	0.69	91 (49.25)	114 (36.75)	0.07	94 (47)	92.5 (55.5)	0.63
**Conjugated bilirubin (mg/dl)**	0.16 ± 0.06 (0.14 - 0.18)	0.18 ± 0.11 (0.15 - 0.21)	0.22	0.17 ± 0.08 (0.14 - 0.2)	0.2 ± 0.11 (0.17 - 0.23)	0.18	0.17 ± 0.06 (0.14 - 0.19)	0.17 ± 0.08 (0.14 - 0.2)	0.81
**Unconjugated bilirubin (mg/dl)**	0.42 ± 0.19 (0.37 - 0.48)	0.42 ± 0.22 (0.36 - 0.48)	0.97	0.44 ± 0.2 (0.37 - 0.5)	0.45 ± 0.19 (0.4 - 0.51)	0.74	0.41 ± 0.16 (0.36 - 0.46)	0.42 ± 0.19 (0.36 - 0.49)	0.7

**ESR (mm/h)**	12 (11.75)	11 (10.25)	0.73	7 (7.25)	13 (12.25)	0.017	10 (11)	7 (9)	0.12
**CRP (mg/L)**	0.9 (2.69)	1.76 (2.62)	0.79	0.5 (1.23)	1.7 (1.46)	0.005	0.7 (2.08)	1.07 (1.47)	0.48
**AST (U/L)**	23.32 ± 8.42 (20.98 - 25.65)	23.25 ± 7.18 (21.29 - 25.2)	0.96	19.25 ± 5.9 (17.41 - 21.08)	23.57 ± 6.87 (21.61 - 25.53)	0.002	19.32 ± 5.65 (17.5 - 21.14)	18.68 ± 5.88 (16.81 - 20.55)	0.63
**ALT (U/L)**	25.72 ± 11.85 (22.43 - 29)	28.32 ± 13.56 (24.64 - 32.01)	0.83	20.1 ± 9.91 (17.02 - 23.17)	28.8 ± 14.09 (24.78 - 32.83)	0.001	19.27 ± 9.33 (16.26 - 22.27)	21.1 ± 10.13 (17.88 - 24.32)	0.41
**Uraemia (mg/dl)**	35.36 ± 12.78 (31.81 - 38.9)	35.17 ± 9.29 (32.64 - 37.69)	0.93	32.33 ± 9.89 (29.26 -35.39)	34.11 ± 7.95 (31.84 - 36.39)	0.35	32.62 ± 9.22 (29.65 - 35.59)	27.86 ± 6.21 (25.89 - 29.84)	0.01
**Serum creatinine (mg/dl)**	0.84 ± 0.15 (0.8 - 0.88)	0.84 ± 0.15 (0.8 - 0.89)	0.95	0.82 ± 0.15 (0.77 - 0.87)	0.87 ± 0.16 (0.82 - 0.92)	0.17	0.83 ± 0.15 (0.78 - 0.87)	0.82 ± 0.15 (0.77 - 0.87)	0.96
**HOMA %B**	102.97 ± 34.11(93.51 - 112.42)	110.95 ± 45.34 (98.62 - 123.27)	0.31	96.1 ± 34.03 (85.55 - 106.65)	114.15 ± 44.53 (101.42 - 126.88)	0.039	92.28 ± 30.13 (82.57 - 101.99	113.44 ± 44.23 (99.37 - 127.5	0.018
**HOMA %S**	76.68 ± 34.65 (67.07 - 86.28)	76.15 ± 40.96 (65.01 - 87.28)	0.94	113.88 ± 54.92 (96.86 - 130.9)	69.78 ± 35.29 (59.69 - 79.87)	< 0.001	103.46 ± 54.02 (86.05 - 120.87	102.18 ± 59.49 (83.27 - 121.1	0.92
**HOMA IR**	1.56 ± 0.65 (1.38 - 1.74)	1.71 ± 0.96 (1.45 - 1.97)	0.36	1.07 ± 0.45 (0.92 - 1.21)	1.8 ± 0.92 (1.54 - 2.06)	< 0.001	1.19 ± 0.48 (1.03 - 1.34	1.28 ± 0.63 (1.08 - 1.48)	0.48
	Anthropometric Variables								
**WC (cm)**	111.68 ± 10.38 (108.8 - 114.55)	112.18 ± 9.6 (109.57 - 114.79)	0.8	103.28 ± 9.77 (100.25 - 106.31)	113.09 ± 8.98 (110.52 - 115.66)	< 0.001	105.07 ± 10.54 (101.68 -108.47)	107.59 ± 8.99 (104.73 -110.45)	0.26
**Weight (kg)**	94.57 ± 14.5 (90.54 - 98.59)	97.07 ± 14.44 (93.15 - 101)	0.38	86.16 ± 12.68 (82.23 - 90.09)	97.07 ± 14.29 (92.98 - 101.16)	< 0.001	88.29 ± 13.62 (83.9 - 92.68)	92.63 ± 14.68 (87.97 - 97.3)	0.18
**BMI (Kg/m^2)**	33.7 ± 4.38 (32.48 - 34.91)	33.87 ± 3.91 (32.8 - 34.93)	0.83	30.89 ± 4.24 (29.57 - 32.2)	33.88 ± 3.44 (32.9 - 34.87)	< 0.001	31.58 ± 4.74 (30.05 - 33.11)	32.14 ± 3.43 (31.05 - 33.24)	0.55
**FM%***	39.72 ± 8.25 (37.43 - 42.01)	40.95 ± 7.32 (38.96 - 42.94)	0.42	28.1 ± 5.32 (26.44 - 29.75)	40.87 ± 7.02 (38.86 - 42.88)	< 0.001	33.79 ± 8.15 (31.16 - 36.42)	33.17 ± 5.76 (31.34 - 35.01)	0.7
**FM (Kg)***	37.41 ± 9.47 (34.78 - 40.04)	39.7 ± 9.11 (37.22 - 42.18)	0.21	24.05 ± 4.91 (22.53 - 25.57)	39.53 ± 8.32 (37.15 - 41.91)	< 0.001	29.79 ± 8.36 (27.1 - 32.49)	30.65 ± 6.76 (28.5 - 32.8)	0.62
**MM%***	26.51 ± 4.33 (25.31 - 27.71)	25.9 ± 4.24 (24.75 - 27.05)	0.47	36.62 ± 7.67 (34.24 - 39)	26.24 ± 4.32 (25 - 27.47)	< 0.001	31.1 ± 5.05 (29.48 - 32.73)	33.2 ± 4.71 (31.7 - 34.7)	0.06
**MM (Kg)***	25.16 ± 5.9 (23.53 - 26.8)	25.22 ± 5.86 (23.62 - 26.81)	0.96	31.7 ± 8.61 (29.03 - 34.37)	25.56 ± 6.07 (23.82 - 27.29)	< 0.001	27.58 ± 6.57 (25.46 - 29.7)	30.72 ± 6.07 (28.79 - 32.65)	0.03
**VFLevel***	15.38 ± 4.93 (14.01 - 16.74)	14.4 ± 4.68 (13.13 - 15.67)	0.3	12.22 ± 4.13 (10.94 - 13.5)	14.4 ± 4.64 (13.07 - 15.73)	0.02	13 ± 4.51 (11.54 - 14.45)	11.94 ± 4.21 (10.6 - 13.28)	0.3

Table S3. Between-group comparisons in biochemical assays and anthropometric measures in all participants at T0, T1 and T2. ALT, alanine aminotransferase; AST, aspartate aminotransferase; TGs, triglycerides; HDL, high density lipoprotein cholesterol; LDL, low-density lipoprotein cholesterol; ESR, erythrocyte sedimentation rate; CRP, C-reactive protein; HOMA %B, steady state beta cell function; HOMA %S, insulin sensitivity; HOMA-IR, homeostasis model assessment of insulin resistance; WC, waist circumference; BMI, body mass index; ng, nanogram; pg, picogram; ml, milliliter; mg, milligram; dl, deciliter; U, international unit; µU, micro international unit; mm, millimiter; L, liter; h, hour; cm, centimeter; m, meter; Kg, kilogram; %, percentage; FM, fat mass; MM, muscle mass; VF, visceral fat. *, estimated by means of bioelectrical impedance analysis. Values are given in mean ± standard deviation (SD) and ± 95% confidence interval (CI) for normally distributed variables or median (interquartile range; IQR) for log-normally distributed variables.

Table S4. Differences in chemosensory testing and biochemical assays in FMD->Control and Control->FMD dropped participants recorded at T0 and T1. Related to "STAR Methods - Supplementary Results – Participants".

	**T0**			**T1**		
	**FMD->Control (n = 10)**	**Control->FMD (n = 5)**		**FMD->Control (n = 3)**	**Control->FMD (n = 9)**	
	**Mean ± SD**	**Mean ± SD)**	**p-value**	**Mean ± SD**	**Mean ± SD**	**p-value**
**OT**	7.15 ± 3.9	8.2 ± 1.95	0.58	8.33 ± 3.32	6.83 ± 1.62	0.3
**OD**	12.5 ± 1.77	13.2 ± 0.83	0.42	12.33 ± 1.15	10.88 ± 1.83	0.23
**OI**	11.9 ± 1.28	12.4 ± 1.51	0.51	12.66 ± 0.57	11.77 ± 0.83	0.12
**TDI**	31.55 ± 4.46	33.8 ± 2.65	0.32	33.33 ± 4.75	29.5 ± 3.1	0.13
**Sweet**	7.1 ± 1.1	5.8 ± 0.83	0.03	7.33 ± 0.57	6.66 ± 1.32	0.42
**Sour**	5.1 ± 1.96	5.6 ± 1.14	0.61	6.66 ± 0.57	5.22 ± 1.39	0.11
**Salty**	6.3 ± 2.11	5.8 ± 2.28	0.68	7.33 ± 0.57	6.22 ± 1.64	0.28
**Bitter**	6 ± 1.76	5.8 ± 1.92	0.84	8 ± 0	6 ± 1.73	0.08
**TTS**	24. 5 ± 4.03	23 ± 2.12	0.45	29.33 ± 1.15	24.11 ± 4.25	0.06
**Leptin (ng/ml)**	29.3 ± 9.15	20.51 ± 2.02	0.05	21.62 ± 6.33	23.37 ± 4.48	0.6
**Ghrelin (pg/ml)**	186.32 ± 38.44	198.19 ± 25.4	0.54	252.97 ± 42.73	201.13 ± 22.2	0.018
**IGF-1 (ng/ml)**	**149.11 ± 14.82**	**183.01 ± 11.96**	**< 0.001**	129.37 ± 10.97	156.19 ± 28.15	0.14
**Insulin (µU/ml)**	10.52 ± 5.15	8.67 ± 1.22	0.45	9.1 ± 5.04	11.47 ± 3.15	0.34

Table S4. Between-group comparisons in chemosensory testing and main biochemical assays in dropped participants recorded at T0 and T1. OT, odor threshold; OD, odor discrimination; OI, odor identification; and their sum (TDI); TTS, total taste score; ng, nanogram; pg, picogram; ml, milliliter; µU, micro international unit. Values are given in mean ± standard deviation (SD). Between-arm comparisons were calculated using two-tailed two-sample t-tests. Bold values indicate statistical significance (set at *p*-value < 0.01) comparisons between FMD->Control and Control->FMD recorded at T0 and T1. Exact p-values are given in the text.
